# Coordinative Stabilization of Single Bismuth Sites in a Carbon–Nitrogen Matrix to Generate Atom‐Efficient Catalysts for Electrochemical Nitrate Reduction to Ammonia

**DOI:** 10.1002/advs.202302623

**Published:** 2023-08-06

**Authors:** Wuyong Zhang, Shaoqi Zhan, Jie Xiao, Tristan Petit, Christopher Schlesiger, Maximilian Mellin, Jan P. Hofmann, Tobias Heil, Riccarda Müller, Kerstin Leopold, Martin Oschatz

**Affiliations:** ^1^ Key Laboratory of Advanced Fuel Cells and Electrolyzers Technology of Zhejiang Province Qianwan Institute of CNITECH Ningbo Institute of Materials Technology and Engineering Chinese Academy of Sciences Ningbo Zhejiang 315201 P. R. China; ^2^ Center for Energy and Environmental Chemistry Jena (CEEC Jena) Institute for Technical Chemistry and Environmental Chemistry Friedrich‐Schiller‐University Jena Philosophenweg 7a 07743 Jena Germany; ^3^ Department of Chemistry‐BMC Uppsala University BMC Box 576 Uppsala S‐751 23 Sweden; ^4^ Department of Chemistry University of Oxford 12 Mansfield Road Oxford OX1 3QZ UK; ^5^ Helmholtz‐Zentrum Berlin für Materialien und Energie GmbH Albert‐Einstein‐Straße 15 12489 Berlin Germany; ^6^ Institute for Optics and Atomic Physics Technische Universität Berlin Hardenbergstr. 36 10623 Berlin Germany; ^7^ Surface Science Laboratory Department of Materials and Earth Sciences Technical University of Darmstadt Otto‐Berndt‐Straße 3 64287 Darmstadt Germany; ^8^ Max Planck Institute of Colloids and Interfaces Department of Colloid Chemistry Am Mühlenberg 1 14476 Potsdam Germany; ^9^ Institute of Analytical and Bioanalytical Chemistry Ulm University Albert‐Einstein‐Allee 11 89081 Ulm Germany

**Keywords:** ammonia production, electrocatalysis, nitrate reduction reaction, single‐site catalysts

## Abstract

Electrochemical nitrate reduction to ammonia powered by renewable electricity is not only a promising alternative to the established energy‐intense and non‐ecofriendly Haber–Bosch reaction for ammonia generation but also a future contributor to the ever‐more important denitrification schemes. Nevertheless, this reaction is still impeded by the lack of understanding for the underlying reaction mechanism on the molecular scale which is necessary for the rational design of active, selective, and stable electrocatalysts. Herein, a novel single‐site bismuth catalyst (Bi‐N‐C) for nitrate electroreduction is reported to produce ammonia with maximum Faradaic efficiency of 88.7% and at a high rate of 1.38 mg h^−1^ mg_cat_^−1^ at −0.35 V versus reversible hydrogen electrode (RHE). The active center (described as BiN_2_C_2_) is uncovered by detailed structural analysis. Coupled density functional theory calculations are applied to analyze the reaction mechanism and potential rate‐limiting steps for nitrate reduction based on the BiN_2_C_2_ model. The findings highlight the importance of model catalysts to utilize the potential of nitrate reduction as a new‐generation nitrogen‐management technology based on the construction of efficient active sites.

## Graphical abstract

A novel synthesis strategy for carbon‐based electrocatalysts with single bismuth sites from metal–organic precursors is reported. Detailed investigations of the materials structures and theoretical calculations provide insights into the structure–property relationships of the catalysts in the electrocatalytic conversion of nitrate to ammonia with particular focus on the influence of the bismuth coordination.

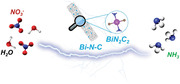

## 1 Introduction

Nitrogen (N_2_), ammonia (NH_3_), and nitrous oxides jointly construct the nitrogen cycle which is critical and fundamental to making life on Earth possible.^[^ ^1^ ^]^ Humankind significantly influences this cycle since the introduction of Haber‐Bosch (H–B) process for artificial nitrogen fixation via the synthesis of ammonia from the elements‐a procedure which has been industrialized more than 100 years ago.^[^ ^2^ ^]^ Industrialization of ammonia production spawned a series of downstream products like fertilizers, explosives, and dyes. Most of these compounds are finally converted to nitrous oxides, which are then mostly present as nitrate. Nature has developed denitrification processes to transfer the nitrate back to dinitrogen and thus to loop the nitrogen cycle.^[^ ^3^ ^]^ Nowadays, the alarming level of nitrate has significantly transformed the natural nitrogen cycle, resulting in a cascade of secondary damages.^[^ ^4^ ^]^ Nitrate contamination of groundwater is widespread over the world and radically changes the diversities of plants and animals.^[^ ^5^ ^]^ Moreover, countless people are exposed to an environment with nitrate concentration above the limits that are seen as critical to lead to non‐Hodgkin's lymphoma and methemoglobinemia.^[^ ^6^ ^]^ Furthermore, nitrogen fixation is still highly dependent on the energy‐intensive H‐B process which is responsible for nearly 2% of global energy consumption and represents around 1% of the total greenhouse gas emissions.^[^ ^7^ ^]^ Novel ammonia production methods that can operate under ambient conditions like the photo/electrochemical dinitrogen reduction reaction (NRR) in aqueous systems are suffering from limited Faradaic efficiency (FE) and poor ammonia formation rates due to the chemical inertness and low water solubility of nitrogen as well as the fierce competition with the hydrogen evolution reaction (HER).^[^ ^8^ ^]^ The direct back‐conversion of nitrate to ammonia is an attractive way to decrease the need for excessive artificial nitrogen fixation and methods to drive this reaction efficiently are in demand.^[^ ^9^ ^]^

To minimize the problems associated with this unbalancing of the natural nitrogen cycle, the electrochemical nitrate reduction reaction (NARR) to ammonia powered by renewable electricity potentially provides a promising solution.^[^ ^10^ ^]^ Nitrate is abundant and can for instance be found in high concentrations in industrial sewage.^[^ ^11^ ^]^ Moreover, NARR takes place at solid–liquid interfaces with a much lower energy barrier than that for solid‐gas‐liquid interfaces as present in NRR with only physically dissolved dinitrogen. This together with the lower dissociation enthalpy of N─O bonds in comparison to N≡N bonds also gives NARR an edge over the competition with HER in comparison to NRR.^[^ ^12^ ^]^ In addition to the development of advanced reactor concepts and the optimization of reaction conditions, the practical implementation of this reaction hinges on the performance of the applied electrocatalysts.^[^ ^13^ ^]^ It is thus imperative to develop catalytically active materials with sufficiently high activity, selectivity, and stability for this emerging field. Nanostructured materials with well‐defined catalytically active centers are especially demanded to illuminate the underlying structure‐activity relationships and gain insights into catalytic mechanisms on a molecular level. Among the various catalytic materials reported for NARR, single‐site catalysts (SSCs) attract special interest due to their often well‐defined active metal centers leading to a maximized atom‐utilization efficiency for the catalytic activation of the nitrate ions. These centers are in most cases stabilized by a carbon matrix containing heteroatoms with electron‐donating properties. On the one hand, the unique performance of SSCs opens up opportunities to develop advanced catalysts for various photo/electrochemical reactions thus addressing the global energy and environmental crisis.^[^ ^13^, ^14^ ^]^ On the other hand, catalytic processes occurring in nature often make use of comparable chemical structure motifs with SSCs surrounded by donor atoms. The defined and tunable coordination environment of such naturally inspired catalysts renders them suitable candidates for the investigation of fundamental mechanisms during catalytic conversions. For instance, just as it is known from fundamental coordination chemistry, the electronic state of metal sites in SSCs and by that also their ability to adsorb and activate small molecules or ions depends on the chemical structure in their surroundings.^[^ ^14a^ ^]^ The development of synthetic approaches towards such model catalytic systems is therefore in demand.

Herein, the development of an SSC featuring porous nitrogen doped carbon decorated with bismuth single sites (denoted as Bi‐N‐C) is reported. The major scope of the work is the investigation of the structure‐property relationships in NARR. Due to their relatively low catalytic activity for the HER, bismuth‐based materials have been widely employed in small molecule activation including nitrate reduction.^[^ ^15^ ^]^ With the introduction of cyanamide as nitrogen source during the pyrolysis process, a bismuth‐based metal‐organic‐material (Bi‐MOM) precursor containing bismuth can be transferred to Bi‐N‐C with metal single sites stabilized at temperatures even up to 1000 °C within a coordination environment that can be described as BiN_2_C_2_. Bi‐N‐C reaches a maximum NARR FE of 88.7% toward ammonia with a corresponding production rate of 1.38 mg h^−1^ mg_cat_^−1^ at −0.35 V versus the reversible hydrogen electrode (RHE). Density functional theory (DFT) calculations provide further insights into the energy profile of NARR on such BiN_2_C_2_ sites which can serve as a guideline for the targeted development of further NARR catalysts and/or other electro‐synthetic processes.

## 2 Results and Discussion

Bi‐MOM was formed by the coordination of Bi^3+^ metal centers by multifunctional 1,3,5‐benzenetricarboxylic acid as the organic linker. This material can be obtained by solvothermal synthesis.^[^ ^16^ ^]^ Scanning electron microscopy shows the nanorod‐like morphology of the rod‐like metal‐organic particles with dimensions in the µm‐range (Figure S1, Supporting Information).

**Figure 1:**
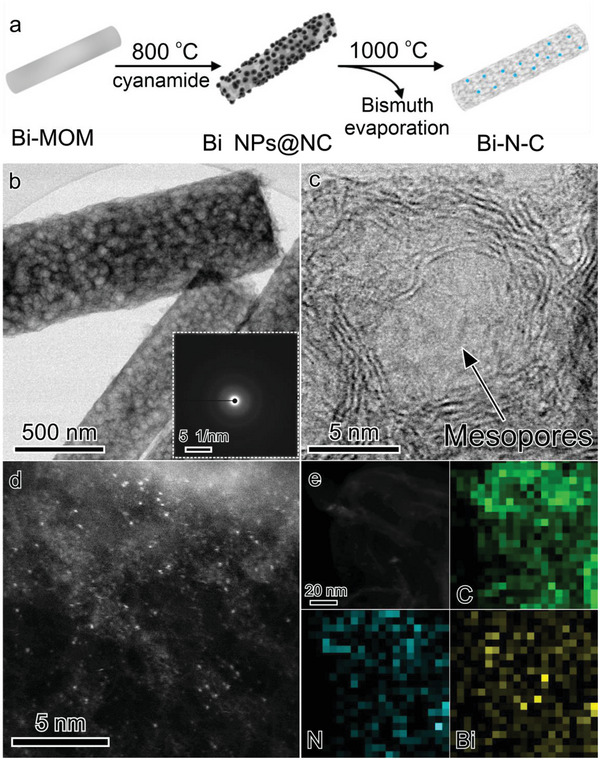
a) Schematic of the synthetic process toward Bi‐N‐C. b) TEM (inset image is the corresponding SAED pattern) and c) HRTEM images of Bi‐N‐C. d) AC HAADF‐STEM image and e) EDX mapping results of Bi‐N‐C.

The Bi‐MOM and cyanamide were placed into a tubular oven within two separate ceramic boats. The cyanamide was positioned in the heated part on the side of the gas flow inlet. Bi‐N‐C formed with the oven temperature raising to 1000 °C (**Figure** **1**a). Thermogravimetric analysis (TGA) of the Bi‐MOM and cyanamide was carried out to investigate the decomposition processes of the precursors (Figure S2a, Supporting Information). The metal–organic precursor pyrolyzes in two major steps. The organic ligand is at first carbonized at around 400 °C and then acts as reducing agent for the transformation of Bi(III) to Bi(0). At the same time, as can be seen from the TGA mass spectrometry results, the pyrolysis of cyanamide produces a series of nitrogen‐containing species like NH_3_ and CN (Figure S2b, Supporting Information), thus leading to N‐doping of the formed carbon network. Hence, during this heating procedure up to 800 °C, a nitrogen‐doped carbon (NC) nanorod structure with encapsulated or deposited Bi nanoparticles was obtained (denoted as Bi NPs@NC). Bi NPs with a broad distribution of sizes and with a surrounding of disordered carbon are clearly observed in transmission electron microscopy (TEM, Figure S3, Supporting Information). At an even higher pyrolysis temperature of 1000 °C, the majority of bismuth evaporates from the material but traces of the metal remain due to strong coordinative interactions with the carbon and doped nitrogen, which results in the formation of a single atom‐decorated material denoted as Bi‐N‐C.^[^ ^17^ ^]^ Due to the sufficiently high vapor pressure of these small bismuth particles and the coordinative stabilization provided by the doped nitrogen and carbon,^[^ ^18^ ^]^ Bi‐N‐C can be obtained by further heating without the need for additional wet chemical etching. This is an advantage in comparison to traditional methods for the preparation of materials containing single metal sites from metal–organic precursors.^[^ ^19^ ^]^ Accordingly, no bulky metallic residuals can be detected in the XRD pattern of Bi‐N‐C. In contrast, metallic bismuth is clearly present in Bi NPs@NC (Figure S4, Supporting Information). The nanorod morphology is conformally transformed from the Bi‐MOM to the Bi‐N‐C (Figure S5, Supporting Information). The obvious removal of Bi NPs during the transformation from Bi NPs@NC to Bi‐N‐C leads to the formation of distinct nanopores as proven by nitrogen physisorption measurements and corresponding pore size analysis (Figure S6, Supporting Information). Compared with the original type I isotherm of the typically microporous parent MOM material, Bi‐N‐C displays a type IV isotherm with a broad hysteresis loop and shows obvious signs of cavitation that is typical for mesoporous adsorbents with bottle‐neck‐shaped pores according to the IUPAC classification.^[^ ^20^ ^]^ The pore size distribution of Bi‐N‐C is rather broad and spans from micropores to mesopores of up to 35 nm. In contrast, Bi‐MOM contains uniform micropores as it is typical for comparable metal–organic materials.^[^ ^21^ ^]^ The corresponding surface area decreases from 749 m^2^ g^−1^ as measured for the parent material to 424 m^2^ g^−1^ for Bi‐N‐C. The open porous structure of Bi‐N‐C was further characterized by TEM and a carbon structure with obvious mesopores can be observed (Figure 1b). This is consistent with the nitrogen physisorption results. The inserted selected area electron diffraction (SAED) pattern taken from the individual nanorod indicates poor crystallinity. Bright‐field high‐resolution TEM (HRTEM, Figure 1c) reveals that the mesopores with a diameter of 5 nm are surrounded by multiple flocculent carbon layers. Furthermore, some dark dots can be observed, which could originate from remaining bismuth species. To elucidate the existence of single bismuth atoms or ions, aberration‐corrected high‐angle annular dark‐field scanning transmission electron microscopy (AC HAADF‐STEM) with sub‐angstrom resolution was employed. Bright dots which could correspond to isolated Bi sites are readily detected (Figure 1d). The bismuth content in Bi‐N‐C was measured to be 1.6 wt% by inductively coupled plasma optical emission spectroscopy (ICP‐OES, Figure S7, Supporting Information). The corresponding energy‐dispersive X‐ray (EDX) elemental mapping demonstrates the homogenous distribution of all elements in Bi‐N‐C (Figure 1e and Figure S8, Supporting Information). The lower signal intensity of bismuth and nitrogen indicates the small content of these elements. The addition of cyanamide as nitrogen source is pivotal to the stabilization of bismuth single atoms. As the nitrogen‐doping process precedes the evaporation of bismuth, the carbon support gets functionalized with N‐sites that can stabilize bismuth at higher temperatures. On the other side, the presence of nitrogen in Bi‐N‐C is related to the existence of bismuth, as nitrogen functionalities will usually also be removed from the carbon framework at high temperatures.^[^ ^22^ ^]^ If cyanamide is absent in the pyrolysis process, a porous carbon material derived from Bi‐MOM can be obtained as well and a nitrogen‐doped carbon (NC) without any metal sites can be fabricated by nitriding it again with cyanamide. NC shows a comparable XRD pattern with Bi‐N‐C, but no indications for the presence of bismuth species can be seen in AC HAAD‐STEM images and in the micro‐X‐ray fluorescence (µXRF) spectra (Figures S9 and S10, Supporting Information). Likewise, µXRF shows that a large amount of bismuth is present in Bi NPs@NC.

**Figure 2:**
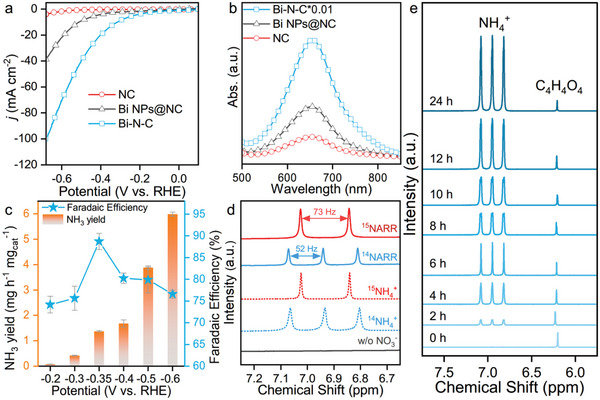
a) LSV curves of Bi‐N‐C, Bi NPs@NC, and NC in 0.5 m KOH + 0.5 m KNO_3_. b) UV–vis spectra of the electrolytes tested with the indophenol blue method after chronoamperometry at −0.35 V versus RHE. c) FE and NH_3_ yield rate of Bi‐N‐C at all given potentials. d) ^1^H NMR spectra of standard samples and electrolytes after NARR with ^14^NO_3_^−^ and ^15^NO_3_^−^ as nitrogen source. e) ^1^H NMR time‐tracking results at −0.35 V versus RHE for Bi‐N‐C.

Based on these structural analyses, Bi‐N‐C, Bi NPs@NC, and NC can be seen as a suitable group of model compounds for the investigation of structure–property relationships of electrochemical NARR with SSCs but also with metal‐free‐ as well as metal nanoparticle‐containing material. The obtained series of materials was therefore subsequently used to illuminate fundamental processes and mechanisms in NARR. Such materials typically have a low catalytic activity towards the HER and are therefore attractive to selectively bind, activate and convert substrates like nitrate in the presence of protons.^[^ ^15^, ^23^ ^]^ Nitrate can strongly bind to bismuth sites, which is beneficial to decrease the energy barriers of the individual reaction steps.^[^ ^15b^ ^]^ Following these considerations, Bi‐N‐C was loaded on carbon paper as a working electrode and dwelled in a H‐cell filled with 0.5 m KOH and 0.5 m KNO_3_ as electrolyte for the NARR tests. Linear sweep voltammetry (LSV) was first performed with Bi‐N‐C, Bi NPs@NC, and NC. Bi‐N‐C exhibits an intensive current response in the presence of nitrate (**Figure** **2**a). In contrast, lower electrochemical activities can be found for Bi NPs@NC and NC. Moreover, as shown by the LSV of Bi‐N‐C without nitrate and the chronoamperometry curve at −0.35 V versus RHE when additional NO_3_^−^ was injected into the electrolyte (initially 0.5 m KOH), Bi‐N‐C showed an intense response to this nitrate addition with a pronounced current increase (Figure S11, Supporting Information). In contrast, NC and Bi NPs@NC showed no obvious current response. As Bi‐based materials are known to be active in NARR to ammonia, the electrolytes of the above three samples were investigated after 5000 s of chronoamperometry at −0.35 V versus RHE with the indophenol blue method.^[^ ^24^ ^]^ Apparently, Bi‐N‐C produced a considerable amount of ammonia in comparison to NC and Bi NPs@NC, as indicated by the intense peak of indophenol blue centered at 655.5 nm in the UV–vis spectra (Figure 2b). Furthermore, the yields of NH_3_ and the corresponding FEs at all given potentials as calculated from the standard curve recorded at different concentrations of NH_4_^+^‐N are plotted (Figure S12, Supporting Information and Figure 2c). Bi‐N‐C produces increasing amounts of ammonia with the cathodic potential getting more negative, reaching the highest rate of 5.99 mg h^−1^ mg_cat_^−1^ at −0.6 V versus RHE. This can also be observed from the corresponding UV–vis spectra of the electrolytes after coloring (Figure S13, Supporting Information). The FEs for ammonia always remain above 70% and reach a maximum of 88.7% with a corresponding ammonia production rate of 1.38 mg h^−1^ mg_cat_^−1^ at −0.35 V versus RHE. With further increasing the applied potential, the ammonia formation rate fails to catch up with the increased current, which leads to the decrease of the FE (Figure 2c and Figure S14, Supporting Information). The ammonia production of Bi‐N‐C was further quantified by ^1^H nuclear magnetic resonance (NMR) spectroscopy (Figure S15, Supporting Information). A FE of 86.7% at −0.35 V versus RHE was calculated, which is comparable to the result obtained from the UV–vis analysis. Nitrite (NO_2_^−^) and hydrogen (H_2_) have been detected as NARR byproducts of Bi‐N‐C, respectively (Figures S16 and S17, Supporting Information). The FEs for NO_2_^−^ are below 5% at all given potentials. The corresponding yield rates increase with a negative potential shift, following the same trend as the ammonia formation. This seems logical as NO_2_^−^ could be an intermediate product during the NARR.^[^ ^25^ ^]^ The FEs for H_2_ are below 20% at all given potentials, which indicates the poor HER activity of Bi‐N‐C. To evaluate the impact of bismuth single sites on the activity of Bi‐based catalytic materials, the ammonia formation rates and FEs of Bi NPs@NC as well as NC were also thoroughly analyzed (Figure S18, Supporting Information). At −0.35 V versus RHE, very limited ammonia was produced over NC. This proves that pristine NC without any bismuth sites has very low catalytic activity in NARR. When employing Bi NPs@NC as the electrocatalyst, a higher production of ammonia than that of NC can be detected. Nevertheless, the FE and ammonia yield rate on Bi NPs are still significantly below the Bi‐N‐C single‐site catalyst. This becomes even more evident considering the significantly higher Bi content in Bi NPs@NC. In addition to the fact that there is no contribution of bismuth atoms that are not on the surfaces of the Bi nanoparticles to catalytic activation of nitrate, the lower electrochemical activity of Bi NPs@NC can also be attributed to the clogging of the pores of the carbon framework by bismuth nanoparticles, which could limit the mass transfer (Figure S19, Supporting Information).^[^ ^26^ ^]^ In contrast, the well‐dispersed and available bismuth single sites within the porous structure of Bi‐N‐C lead to superior FE and ammonia yield. Otherwise, compared with other reported catalysts (Table S1, Supporting Information), Bi‐N‐C shows promising FE with decent current density under low overpotential.

To confirm the source of nitrogen within the produced ammonia, ^15^N isotopic labelling experiments were employed and the product was characterized with ^1^H NMR (Figure 2d). ^15^N is a spin‐½ nucleus that will result in two symmetric signals for ^1^H‐^15^N, but three symmetric signals will arise for ^1^H‐^14^N with spin‐1 nucleus.^[^ ^27^ ^]^ With the standard ^14^NH_4_^+^ and ^15^NH_4_^+^ samples as references, it can be clearly observed that the electrolytes after NARR with ^15^NO_3_^−^ exhibited the typical double peaks of ^15^NH_4_^+^ at the chemical shifts of 6.84 and 7.02 ppm with a spacing of 73 Hz. This is in contrast to the triple peaks of ^14^NH_4_^+^ that are present after NARR with ^14^NO_3_^−^ at chemical shifts of 6.85, 6.94, and 7.07 ppm with a spacing of 52 Hz. If NO_3_^−^ is absent, no ammonia signal is present in the ^1^H NMR spectrum. Quantification of the FEs and ammonia formation rates between ^15^NO_3_^−^ and ^14^NO_3_^−^ based on UV–vis spectroscopy show only minor differences at an adopted electrolyte composition (Figure S20, Supporting Information).

The stability of Bi‐N‐C has also been studied as this is another essential measure for its application as a catalyst. During 24 h of catalyst operation, no obvious decay in the activity of Bi‐N‐C could be observed as indicated by the rather stable current in the chronoamperometry curve (Figure S21a, Supporting Information). The corresponding UV–vis spectra of the diluted electrolyte samples over electrolysis time confirm the stable NARR activity of Bi‐N‐C (Figure S21b,c, Supporting Information). The slight decrease of the ammonia formation rate after 12 h of operation can at least in parts be attributed to the crossover of ammonium driven by the concentration gradient built up between the two electrode compartments of the H‐cell (Figure S22, Supporting Information). The quantification of species dissolved in the electrolyte by ^1^H NMR also manifests the high activity and stability of Bi‐N‐C during the 24 h test (Figure 2e). Moreover, the Bi‐N‐C cathode was coupled with an anode of nickel–iron layered double hydroxide (Ni‐Fe LDH, Figure S23, Supporting Information) on nickel foam to construct a device. The voltage of the two‐electrode system (Bi‐N‐C||Ni‐Fe LDH) required to reach 100 mA cm^−2^ remained almost stable over 500 h (Figure S24, Supporting Information). Bi‐N‐C maintained the original morphology after the stability test in TEM and single sites still remained without any larger clusters or nanoparticles that can be observed in AC HAADF‐STEM, the given elements in Bi‐N‐C also kept a homogenous distribution (Figure S25, Supporting Information).

**Figure 3:**
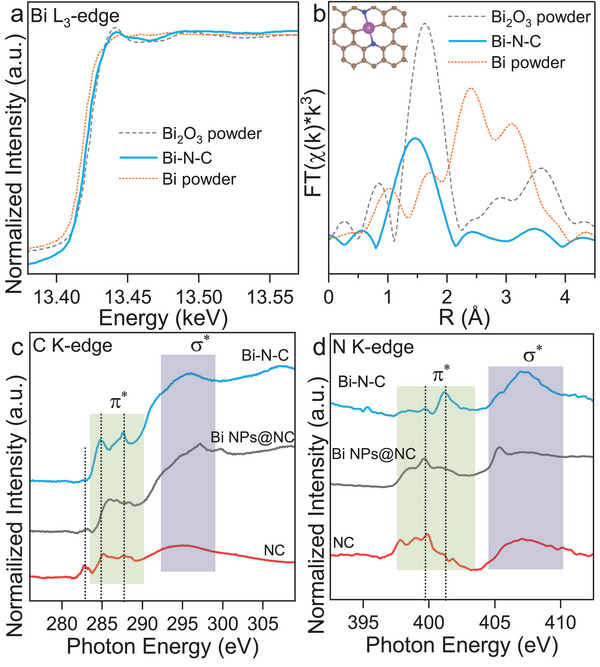
Bi L_3_‐edge a) XANES and b) EXAFS spectra of Bi‐N‐C, Bi_2_O_3_ powder, and Bi powder (the inset shows the model of the Bi coordination in Bi‐N‐C with Bi (purple), N (blue), and C (brown)). c) C K‐edge and d) N K‐edge XANES spectra of Bi‐N‐C, NC, and Bi NPs@NC (the baseline of NC in the N K‐edge spectrum has been subtracted here).

In order to illuminate the origin of the high catalytic activity and the underlying reaction mechanism, the structure of the active sites in Bi‐N‐C has been analyzed in more detail using various X‐ray spectroscopy‐based techniques. X‐ray photoelectron spectroscopy (XPS) measurements show that the bismuth signal is almost invisible due to the low atomic metal content in Bi‐N‐C (Figure S26b, Supporting Information). The high‐resolution carbon C 1s and N 1s core level spectra indicate that the carbon mostly consists of graphitic sp^2^ carbon with some remaining C═N bonds with nitrogen mostly bonded as graphitic nitrogen (Figure S26c,d, Supporting Information). The electronic structure of Bi‐N‐C was further confirmed by the white‐line intensity between Bi and Bi_2_O_3_ powder under X‐ray absorption near‐edge structure (XANES) examination (**Figure** **3**a). The results indicate that the metal in Bi‐N‐C is present in a valence state of Bi*^δ^* ^+^ (0<*δ*<3). In extended X‐ray absorption fine structure (EXAFS) analysis (Figure 3b), Bi‐N‐C shows a main peak at about 1.44 Å, which could be attributed to the Bi‐N or Bi‐C scattering path. Moreover, no intense Bi–Bi scattering path at about 3.09 Å can be observed for Bi‐N‐C, demonstrating that the as‐prepared Bi‐N‐C contains isolated Bi species coordinatively stabilized by the N‐doped carbon surrounding. The chemical environment of bismuth centers was further confirmed by virtue of soft X‐ray absorption spectroscopy (sXAS) as this method is sensitive to the local configuration around the probed element. As indicated by the carbon K‐edge XANES (Figure 3c), Bi‐N‐C shows a sharp peak at 287 eV with a negative shift at the π* transition region relative to NC and Bi NPs@NC. This indicates the strong chemical interaction resulting from the stabilization of bismuth by the nitrogen‐doped carbon surrounding.^[^ ^28^ ^]^ The sharp peaks in Bi‐N‐C might be attributed to the π* transition in C─Bi bonds, which are not observed in Bi NPs@NC and NC. Notably, the peak at 282.7 eV of NC can be assigned to carbon defects formed by the removal of nitrogen at a high temperature of 1000 °C,^[^ ^29^ ^]^ which is consistent with the higher *I*_D_/*I*_G_ of NC as compared to Bi‐N‐C in Raman spectroscopy (Figure S27, Supporting Information). The absence of nitrogen also results in fuzzy resonances in nitrogen K‐edge XANES and no obvious signal in the XPS survey spectrum of NC (Figure 3d and Figure S28, Supporting Information). However, due to the stable coordination with bismuth, nitrogen in Bi‐N‐C is still present and shows two typical spectroscopic features: π*_C‐N‐C_ (401.1 eV) and σ*_C‐N‐C_ (405 eV to 410 eV).^[^ ^30^ ^]^ Compared with Bi NPs@NC which contains more nitrogen with pyrrolic‐N dominating (Figure S29, Supporting Information), the π*_C‐N‐C_ peak of Bi‐N‐C has a positive shift which can be attributed to the chemical bonding with bismuth. Based on these results, it can be concluded that during the pyrolysis process of the Bi‐MOM, bismuth, and nitrogen remained in the materials and stabilized each other. EXAFS fitting (Figure S30, Supporting Information) suggests that the atomic construction of Bi‐N‐C can be described as individual bismuth centers coordinated by two nitrogen atoms and two carbon atoms (BiN_2_C_2_) with a coordination number of 4 (Table S2, Supporting Information).

Based on these results, the BiN_2_C_2_ motif was used as a starting model for theoretical calculations and has been optimized and investigated for its NARR mechanism as the active site. The symmetrical BiN_2_C_2_ model (Figure S31a, Supporting Information) has a Bi‐C bond length of 2.24 Å and a Bi─N bond length of 2.30 Å. This is around 0.06 Å shorter than the Bi─N bond length in the second possible configuration (Figure S31b, Supporting Information), which results in a 0.11 eV lower electronic energy. Bader charge analysis shows that the charge of the Bi site in BiN_2_C_2_ is 0.933e, transferring charge to the bonded C (accounting for 21% of the 0.933e) and N (accounting for 79%) atoms.

**Figure 4:**
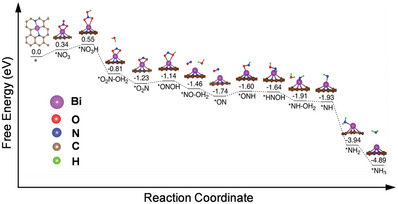
The energy panel of the minimum energy pathway on the BiN_2_C_2_ model.

The reasons behind the high activity of Bi‐N‐C for NARR as well as the underlying reaction mechanisms were further illuminated with DFT calculations based on the assumption of a BiN_2_C_2_ structure model for the active sites and a *NO_3_ radical molecule as nitrogen oxide model compound. Nitrate reduction to ammonia is accompanied by 9‐proton and 8‐electron transfers and involves many intermediates.^[^ ^31^ ^]^ Different possible reaction pathways to form products on BiN_2_C_2_ were investigated (Figure S32, Supporting Information). We are reminding the readers that calculations of suchlike energy profiles do actually not consider the formation and structure of electric double‐layers or the influence of local pH fluctuations which are likely to be dominant under reaction conditions at the interfaces formed between porous electrode materials and electrolytes. Although also no activation barriers are calculated here, the most reasonable reaction pathways for the considerable BiN_2_C_2_ and BiN_4_ active sites can be estimated from such calculated energy profiles. One possible energy profile (**Figure** **4**) shows that the *NO_3_ adsorption on the BiN_2_C_2_ is unfavorable with the highest uphill free energy of 0.34 eV during the entire catalytic process. This initial energy demand is in good accordance with the experimentally observed onset potential in the range of −0.2 to –0.3 V versus RHE. The first hydrogenation on the *NO_3_ (*NO_3_ to HNO_3_) without electron transfer also shows an uphill free energy of 0.21 eV.^[^ ^25^ ^]^ The reduction pathways of HNO_3_* to NO* on BiN_2_C_2_ are nearly downhill in free energy with two different binding modes, except one hydrogenation step on NO_2_* to *ONOH with a slight uphill free energy of 0.09 eV. The NO* has been suggested as a key intermediate in the previous studies on nitrate reduction on metal surfaces or SSCs which is in agreement with the computational results on BiN_2_C_2_.^[^ ^32^ ^]^ Formation of NO* reaches a local minimum in the entire process, followed by hydrogenation on the nitrogen atom (instead of the oxygen atom) with an uphill free energy of 0.14 eV. On the contrary, the pathway of hydrogenation on the oxygen atom (*NO to *NOH) is unfavorable with a 1.08 eV uphill free energy (Figure S33, Supporting Information). The subsequent reactions to form NH_3_ (after the release of one H_2_O molecule, *NH‐H_2_O to *NH) are all exergonic. The successive exothermic reaction steps contribute to the remarkable catalytic performance of the BiN_2_C_2_. BiN_4_ is selected as a reference as it can also be a reasonable model only with the EXAFS fitting (Table S2, Supporting Information). Compared with BiN_2_C_2_, the charge of the Bi site is 1.178e as calculated from Bader charge analysis. This results in stronger binding affinity of *NO_3_ adsorption with a downhill free energy of 0.87 eV. However, the higher charge of the Bi center in BiN_4_ seems unfavorable for stabilizing hydrogenated intermediates, which is reflected in the uphill free energy of 0.7 eV for the protonation of *NO_3_ (the potential limiting step for the pathway with BiN_4_, Figure S34, Supporting Information), hydrogenation on NO_2_* and NO* with an uphill free energy of 0.5 eV. As experimental results have shown that the local sites of Bi‐N‐C can be best described as BiN_2_C_2_, the combination of the experimental results and DFT calculations, allows for the conclusion that the high NH_3_ yield rate can be attributed to the intrinsically high‐efficiency active sites, that is, the lower charge of the Bi centers on the BiN_2_C_2_.

## 3 Conclusion

In summary, a nitrogen‐doped carbon catalyst decorated with bismuth single sites has been synthesized by pyrolyzing a Bi‐MOM precursor in the presence of cyanamide at 1000 °C. Due to the mutual coordinative stabilization between bismuth and the support material with donor functions, traces of bismuth and nitrogen are still present even after the high‐temperature treatment. X‐ray spectroscopy measurements revealed that the coordination of bismuth is best described as BiN_2_C_2_. Thanks to the high bismuth utilization, Bi‐N‐C exhibits remarkable activity and selectivity in the electrochemical nitrate reduction to ammonia even at very low metal content. The possible mechanisms and reaction pathways of NARR on such BiN_2_C_2_ are further studied by DFT calculations. It is found that the Bi center in BiN_2_C_2_ has a relatively low charge. The low‐charge Bi center results in the unfavorable adsorption of nitrate but is beneficial to stabilize the hydrogenated intermediates. This work provides fundamental insights into the working mechanisms of Bi‐based active centers for ammonia synthesis from the back‐conversion of nitrate with electrochemistry.

## Conflict of Interest

The authors declare no conflict of interest.

## Supporting information

Supporting Information

## Data Availability

The data that support the findings of this study are available from the corresponding author upon reasonable request.
