# Supplementary material for: Coordinative Stabilization of Single Bismuth Sites in a Carbon–Nitrogen Matrix to Generate Atom‐Efficient Catalysts for Electrochemical Nitrate Reduction to Ammonia
Source: Adv Sci (Weinh). 2023 Aug 6;10(28):2302623. doi: 10.1002/advs.202302623 (PMC10558634; doi:10.1002/advs.202302623)
Supplement: Supplementary file 1 — Supporting Information [file ADVS-10-2302623-s001.pdf]

ADVANCED SCIENCE
Open Access

Supporting Information

for *Adv. Sci.*, DOI 10.1002/advs.202302623

Coordinative Stabilization of Single Bismuth Sites in a Carbon–Nitrogen Matrix to Generate Atom-Efficient Catalysts for Electrochemical Nitrate Reduction to Ammonia

Wuyong Zhang, Shaoqi Zhan, Jie Xiao, Tristan Petit, Christopher Schlesiger, Maximilian Mellin, Jan P. Hofmann, Tobias Heil, Riccarda Müller, Kerstin Leopold and Martin Oschatz**

Supporting Information

Coordinative Stabilization of Single Bismuth Sites in a Carbon-Nitrogen Matrix to Generate Atom-Efficient Catalysts for Electrochemical Nitrate Reduction to Ammonia

Wuyong Zhang, Shaoqi Zhan, Jie Xiao, Tristan Petit, Christopher Schlesiger, Maximilian Mellin, Jan P. Hofmann, Tobias Heil, Riccarda Müller, Kerstin Leopold, and Martin Oschatz**

Experimental details

Reagents: Bismuth nitrate pentahydrate ($Bi(NO_3)_3·5\ H_2O$, 99%), anhydrous methanol (CH_3OH, 99.9%), 1,3,5-benzenetricarboxylic acid (H_3BTC), cyanamide (CH_2N_2, 99.9%), potassium hydroxide (KOH, 99.8%), potassium nitrate ($K^{14}NO_3$, $K^{15}NO_3$ 99.9%), diaminomaleonitrile salicylic acid ($C_7H_6O_3$, 99.9%), potassium sodium tartrate ($NaKC_4H_4O_6·4H_2O$, 99.9%), ammonium chloride (NH_4Cl, 99.98%), hydrochloric acid (HCl, 99.999% metal base), sodium hypochlorite (NaClO, available chlorine 6-14 %), para-(dimethylamino) benzaldehyde ($C_9H_{11}NO$, 99.9%) were purchased from Alfa Aesar. Standard NH_4^+-N solution (6 mg L^{-1}) and NO_2^--N solution (40 mg L^{-1}) were purchased from Merck. Iron nitrate nonahydrate ($Fe(NO_3)_3·9H_2O$, 99.99%) and nickel nitrate hexahydrate ($Ni(NO_3)_3·6H_2O$, 99.99%) were purchased from Sinopharm Chemical Reagent. Unless otherwise stated, ultrapure (18.2 MΩ·cm) water was used in all experiments. All chemicals were used without further purification.

Synthesis of Bi-MOM: Bi-MOM was prepared by a solvothermal method reported in the literature.[1] In a typical solvothermal preparation process, H_3BTC (1 g) was firstly dissolved in 40 mL anhydrous methanol at room temperature, and $Bi(NO_3)_2·5\ H_2O$ (83.3 mg) was added to the above solution. Then the mixed solu-

tion was transferred into a 60 mL Teflon-lined stainless-steel autoclave. The autoclave was heated to 120 °C and hold for 24 h. The obtained white precipitates were collected by centrifugation, washed three times with anhydrous methanol, and finally dried in an oven at 80 °C overnight for further use without any additional processing.

Synthesis of Bi-N-C: As-prepared Bi-MOM (120 mg) and cyanamide (600 mg) were placed at two separate positions in the heated zone of a tube furnace and heated to 1000 °C (heating rate 2 °C min^{-1}) for 3 h in a stream of Ar (10 mL min^{-1}) to produce the Bi-N-C material.

Synthesis of Bi NPs@NC: The similar synthesis process as for Bi-N-C has been applied but the maximum pyrolysis temperature was set to 800 ^{o}C.

Synthesis of NC: NC was obtained by a two-step process. The first step was similar to the synthesis of Bi-N-C but with the absence of cyanamide to get a carbon without metal sites. In the second step, is carbon and cyanamide (600 mg) were placed into separate positions in the heated zone of a tube furnace and heated to 1000 °C (heating rate 2 °C min^{-1}) for 3 h in a stream of Ar (10 mL min^{-1}) to get NC.

Synthesis of NiFe-LDH: The nickel-iron layered double hydroxide (Ni-Fe LDH) nanosheet arrays were synthesized through a typical solvothermal method. 0.5 mmol $Ni(NO_3)_2 \cdot 6H_2O$ and 0.5 mmol $Fe(NO_3)_3 \cdot 9H_2O$ and 10 mmol urea were dissolved in 35 mL water and stirred to form a clear solution. Four pieces of Ni foam (about 1 cm × 4 cm) and the solution were transferred to a 50 mL Teflon-lined stainless steel autoclave and maintained at 120 ℃ for 12 hours. After naturally cooling to room temperature, the NiFe-LDH was rinsed with water and ethanol for 3 min to remove residual ions, and then dried at 80 ℃ overnight for further use.

Characterizations:

Scanning electron microscopy (SEM) was carried out on a LEO 1550-Gemini microscope operating at 3.00 kV. A thin platinum layer of a few nanometers thickness was sputter onto the surface of the samples to increase the surface conductivity. **Energy-dispersive X-ray (EDX)** investigations were conducted using a Link ISIS-300 system (Oxford Microanalysis Group) equipped with a Si (Li) detector and an energy resolution of 133 eV.

Transmission electron microscopy (TEM) and high-resolution transmission electron microscopy (HRTEM) was performed on a JEOL ARM 200F microscope at an acceleration voltage of 200 kV. **The aberration-corrected high-angle annular dark-field scanning transmission electron microscopy (AC HAADF-STEM)** images were acquired using a double-Cs-corrected JEOL ARM 200F, equipped with a cold field emission gun and an acceleration voltage set to 80 kV. The microscope is further equipped with a JED-2300 energy-dispersive X-ray detector, which was utilized for the EDX measurements.

Powder X-ray diffraction (PXRD) patterns were recorded on a Bruker D8 Advance diffractometer equipped with a scintillation counter detector with Cu Kα radiation (λ = 0.15184 nm) applying 2θ step size of 0.025°.

X-ray photoelectron spectroscopy (XPS) data were obtained on a Physical Electronics spectrometer in fixed analyzer transmission mode using monochromatic Al K_α radiation ($h\upsilon$ = 1486.6 eV, spot diameter 200 µm and a power of 50 W) at an angle of 45° with a pass energy of 23.50 eV (step size of 0.1 eV) for region scans. The samples have been measured on indium foils and due to the good conductivity, no charge neutralization has been applied.

Micro X-ray fluorescence (µXRF) spectra were recorded using an M4 Tornado (Bruker Nano GmbH, Berlin, Germany) equipped with a Rh X-ray tube which was operated at maximum power (50 kV, 600 µA), polycapillary optics that focus X-ray excitation radiation to a spot of approximately 25 µm, and an XFlash 430-PA detector for X-ray fluorescence with an active area of 30 mm^2 providing a resolution of <145 eV at Mn K_{α}. Spectra were recorded without application of an energy filter.

Direct analysis of the powder samples Bi-N-C, Bi NPs@NC and blank NC, was performed after filling the solid sample into a cavity of approx. 40 mm^3 of a polymethyl methacrylate (PMMA, diameter 30 mm, thickness 3mm; Bruker Nano GmbH) sample carrier covered by X-ray transparent Ultralene® foil (SPEX Sample Prep, Metuchen, NJ, USA) which was fixed using double sided adhesive carbon tape (Spectro-Tabs, Plano GmbH, Wetzlar, Germany). This house-made sample holder was attached to the M4 Tornado sample stage and allows interference-free measurement under reduced pressure at 20 mbar. Six random sampling positions were selected for every sample, i.e. 6 point measurements were recorded with a lifetime of 500 s. OriginPro 2021b (9.8.5.201) was used for the baseline correction and peak integration. For the baseline correction the asymmetric least square smoothing was used with an asymmetric factor of 0.001, a threshold of 0.025, a smoothing factor of 7, and number of iterations was set to 10.

X-ray absorption spectroscopy (XAS) measurements were carried out with a novel self-developed wavelength-dispersive spectrometer in von Hámos geometry.[2] The spectrometer is equipped with a microfocus X-ray tube (rtw MCBI 50B-70 Mo optimized to 15 kV), a curved highly annealed pyrolytic graphite mosaic crystal and a hybrid photon counting CMOS detector with 512 × 1030 pixels and a pixel size of 75 µm × 75 µm (DECTRIS EIGER2 R 500K). The tube

was operated with a voltage and current of 19.8 kV and 1500 μA for Bi LIII-edge. All references and samples were in powder form and prepared as wax-pellets (mixed with Hoechst Wax C), due to their low concentration of Bi. A pellet with a 13 mm diameter was pressed by using a hydraulic pellet press with force up to 6 tons for not longer than 60 s. As the samples were measured in transmission mode, the absorption spectrum was acquired by measuring once with and once without the sample. The measurement time for each sample varied between 5 and 15 h depending on the thickness of the prepared sample. All references and samples were constantly moved during the measurements to minimize the effects of local thickness inhomogeneity. The beam size on the samples is around 3 mm × 3 mm. Normalization of the spectra was done by using the XAS analyzing and processing software ATHENA which is part of the Demeter software package.[3] The obtained XAS original data was processed for the background, pre-edge line, post-edge line correction and normalized in Athena. The EXAFS fitting was conducted in Artemis. A k range of 2-11 $Å^{-1}$ and k-weighting of 2 were used for all the Bismuth-samples. The amplitude reduction factor (S_0^2) was determined to be 0.81 (single scattering fitting, R range 1.0-3.0 Å), which was used for all the other Bi-data fitting process.

The soft X-ray absorption spectroscopy (sXAS) for nitrogen and carbon were acquired in total electron yield (TEY) mode on the catalyst material deposited on a conductive copper tape. The measurements were performed at the U49/2 PGM-1 beamline of the synchrotron BESSY II using the LiXEdrom endstation.

N_2 physisorption experiments were performed at -196 °C on a Quadrasorb apparatus (Quantachrome Instruments, USA). Prior to all the physisorption measurements, the samples were outgassed under vacuum at 140 °C for overnight. Specific surface areas (SSA) of the materials are calculated by using the multipoint Brunau-

er-Emmett-Teller (BET) model in the relative pressure range 0.05-0.2. The total pore volumes (V_t) were determined at $p/p_0 = 0.99$. The pore size distributions are calculated by using the quenched solid density functional theory (QSDFT) method for nitrogen on carbon with slit/cylindrical/spherical pores at -196 °C, adsorption branch kernel, integrated into the QuadraWin 5.11 analysis software (Quantachrome).

Thermal Gravimetric Analysis (TGA) and **TGA mass spectrometry (TGA-MS)** were collected on a thermo microbalance TG 209 F1 Libra (Netzsch, Selb, Germany) coupled with a Thermostar Mass spectrometer (Pfeiffer Vacuum; Asslar/Germany) with an ionization energy of 75 eV. A platinum crucible was used for the measurement of 10 ± 1 mg of samples in a N_2 flow of 10 mL min^{-1} and a purge flow of 10 mL min^{-1}. The samples were heated to 1000 °C with a heating rate of 5 K min^{-1}. The data was recorded and analyzed by the Proteus (6.0.0) and Quadstar (7.03) software package. TGA-MS measurement was performed using helium with a flow of 10 ml min^{-1} and with a heating rate of 2.5 K min^{-1}.

Raman spectra were recorded using a Witec (focus innovations) Raman Microscope operating with an objective (Nikon, 50x/0.25, ∞/- WD 6.1) and an excitation wavelength of 532 nm with an intensity of 1 mW, the integral time is 30s for three cycles. The Raman fitting is following the four-peak model proposed by Sadezky et al. for graphitized carbon materials.[4]

Nuclear Magnetic Resonance (NMR) spectra were obtained on a Bruker AVANCE III 600 MHz.

UV-vis spectra have been measured on a UV/VIS Spectrometer (Lambda 750, Perkin-Elmer). The scan rate and slit width were set to 120 nm min^{-1} and 0.5 mm, respectively.

Gas chromatography (GC) measurements were obtained on Agilent GC (7890B) with a with a thermal conductivity detector (TCD) for H_2. The partial current density of H_2 (j_{H_2}) can be calculated by the following equation:

$$j_{H_2}=\text{flow rate}*\frac{2F*x}{V_m}*(\text{electrode area})^{-1}$$

Where the x is concentration of gaseous products (mol mol^{-1}), flow rate is 40 mL min^{-1} ($6.67*10^{-4}$ L s^{-1}), F is the faraday constant of 96485 C mol^{-1}, V_m is the molar volume of gas (22.4 L mol^{-1}). With this, the Faradaic Efficiency for hydrogen (FE_{H_2}) can be calculated by $FE_{H_2}=\frac{j_{H_2}}{j_{total}}$.

Electrochemical measurements:

Prior to the measurements, the working electrodes have been prepared by depositing a dispersion of the catalyst in ethanol (1 mg/mL, 20 μL Nafion solution contained) on carbon paper (1*1 cm^2) with a nominal mass loading of 1 mg cm^{-2}. The measurements were conducted on an Autolab electrochemical setup (PGSTAT204, Metrohm) with a three-electrode system. The counter electrode and reference electrode were platinum foil (1*1 cm^2) and a saturated calomel electrode (SCE), respectively. All potentials in this work are referred to RHE via the correction (0.059*pH+0.242) V. 0.5 M KOH and 0.5 M KOH + 0.5M KNO_3 aqueous solutions were prepared with ultrapure water from Millipore system and used as electrolytes. Electrolysis was performed in a H-type cell separated by Nafion membrane. Prior to the test, the Nafion membrane was pretreated by heating in 0.5 M H_2SO_4, ultrapure water, 5% H_2O_2 aqueous solution, and ultrapure water at 80 °C for 30 mins in turn. Linear sweep voltammetry (LSV) tests were conducted in Ar-saturated solution with a scan rate of 1 mV s^{-1}, the purity of the gas used in all experiments is 99.999%. The potentiostatic test was carried out at different potentials

for 2 h. FE of the ammonia formation was calculated from the percentage of the total amount of the charge consumed for the production of ammonia (n_{NH_3}) in the total charge Q (C) passed through the electrochemical system. Since eight electrons are transferred for the formation of one ammonia molecule with dinitrogen molecule, the FE can be calculated as follows: FE= n_{NH_3}*8F/(I*t), where the F is the Faraday constant (96485.34 C mol^{-1}), I (A) is the current at the potential applied on the electrochemical system, and t (s) is the electrolysis time. The FE of the possible nitrite byproduct has been calculated in the same way but with counting two transferred electrons. The nitrate-response chrono-amperometry experiments were performed by injecting a concentrated nitrate solution with volume of 1 mL into the blank solution of 0.5 M KOH with -0.35 V applied over NC, Bi NPs@NC and Bi-N-C.

Detection of NARR products:

The concentrations of ammonium and nitrite in the electrolyte were analyzed with UV-vis spectroscopy and quantified based on the recorded standard curves. Before testing, the electrolyte was diluted in different ratios for some samples to fit the UV-vis calibration range depending on the concentration of ammonium/nitrite in the electrolyte after reaction.

For ammonium,[5] 2 mL diluted electrolyte was mixed with 2 mL chromogenic reagent of 1 M NaOH solution (containing 5 wt% of salicylic acid and 5 wt% of sodium citrate), followed by adding 1 mL oxidizing solution of 0.05 M NaClO and 0.2 mL catalyzing reagent of 1 wt% sodium nitroferricyanide. After standing at room temperature for 1 h, the produced indophenol blue was detected by UV-vis spectroscopy. The standard curve was plotted with the absorption intensity at 655.5 nm and the concentration of standard NH_4^+ solution. (The dilution ratios for given potentials: (-0.2 V:5, -0.3 V:20, -0.35 V:100, -0.4 V:100, -0.5 V: 200, -0.6 V: 400).

The ammonia production is also proved by ^{1}H NMR with the internal standard method. Maleic acid ($C_4H_4O_4$) was selected as the internal standard with DMSO-d_6 as the deuterated solvent (20 mg $C_4H_4O_4$ dissolved in 50 g DMSO-d_6). Before the test, a series of NH_4^+ solution (450 μL, 0.5 M KNO_3+0.5 M KCl, pH=2) with different concentration was prepared and mixed with DMSO-d_6 (100 μL with internal standard inside) for the NMR test. The calibration curve was plotted by the peak integral area of NH_4^+ and corresponding concentrations. It is worth mentioning that the pH of solution should be adjusted to two with concentrated hydrochloric acid before test and the test process of NARR samples is same with that for recording the calibration curve.[6] The ammonium concentration in the NARR electrolyte sample can then be calculated by the calibration curve.

For nitrite,[7] 0.2 g of N-(1-naphthyl) ethylenediamine dihydrochloride, 4 g of p-aminobenzenesulfonamide, and 10 mL of phosphoric acid (ρ = 1.685 g mL^{-1}) were added into 50 mL of deionized water and mixed thoroughly as the coloring reagent. 5 mL of the diluted electrolyte which fits the UV-vis testing range and 0.1 mL of color reagent were mixed together. After 20 min at room temperature, the UV-vis absorption spectrum was measured and the absorption intensity was recorded at a wavelength of 500 nm. A series of standard NO_2^--N solutions were used to obtain the concentration-absorbance curve by the same processes. (The dilution ratios for given potentials: (-0.2 V:2, -0.3 V:2, -0.35 V:10, -0.4 V:10, -0.5 V: 10, -0.6 V: 10).

Isotope Labeling Experiments were performed with $K^{15}NO_3$ as N-source for the potentiostatic test. After electrolysis, the pH of electrolyte was adjusted to two by concentered HCl for further analysis by ^{1}H NMR (600 MHz).

Computational details:

All the DFT calculations were performed by the Vienna ab initio simulation package (VASP).[8] The electron-core interactions were described by the projected

augmented wave (PAW)[9] method and electron exchange-correlation was expressed at the general gradient-approximation (GGA) level by the Perdew–Burke–Ernzerhof (PBE) functional.[10] A cutoff energy of 500 eV was adopted for the plane-wave basis set. Spin polarization was used in all calculations. For structural optimization, a convergence threshold of 0.03 eV $Å^{-1}$ was set in force and the total energy converged to within 10^{-5} eV. Grimme's method (DFT-D3)[11] was included during the surface adsorption to better evaluate the van der Waals' interaction. The BiN_4 and BiN_2C_2 models were constructed by enclosing a Bi atom with one layer of 4 × 4 graphene,

where four and two C atoms were replaced by N atoms, respectively. The Gamma-point was considered for sampling the Brillouin zone during the calculations. In the surfaces, there was a vacuum region of 15 Å in the z direction. For integration over the reciprocal space, we used a 3 × 3 × 2 k-point mesh with horizontal shifts. Bader charge analysis was used to obtain the partial atomic charges of the BiN_2C_2 and BiN_4 model. The standard hydrogen electrode (SHE) model proposed by Nørskov, in which the chemical potential of a proton-coupled-electron pair is equal to half of the chemical potential of H_2, was used to calculate the Gibbs free energy (ΔG). Therefore, the ΔG of all nitrate reduction reactions (NARR) were computed by $\Delta G = \Delta E + \Delta ZPE - T\Delta S$, where ΔE is the electronic energy difference, ΔZPE is the change of zero-point energy, and ΔS is the change of the entropy. The ZPE and entropies S were calculated by the vibrational frequencies of all species in which only vibrational mode of the adsorbed species are computed explicitly and the surfaces are fixed. VESTA was used to draw the molecular structure. All the computational results are obtained under the conditions of 0 V and vacuum.

Supplementary Figures

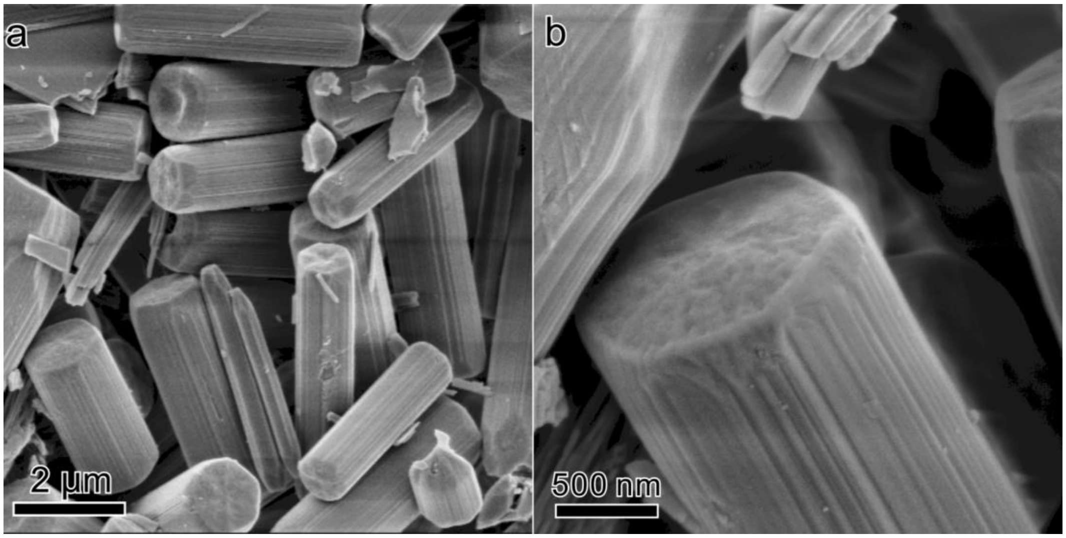

Figure S1. (a) SEM images of Bi-MOM.

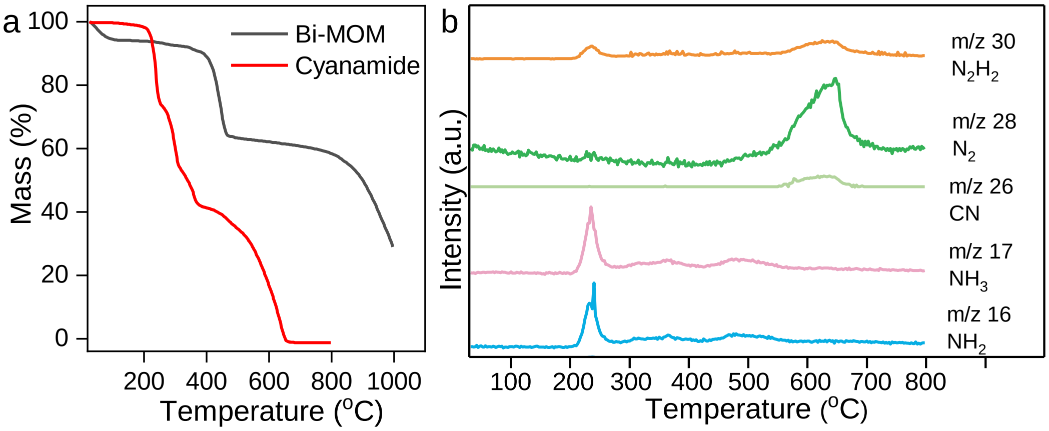

Figure S2. (a) TGA curves of Bi-MOM and cyanamide and (b) corresponding TGA-MS curves of cyanamide.

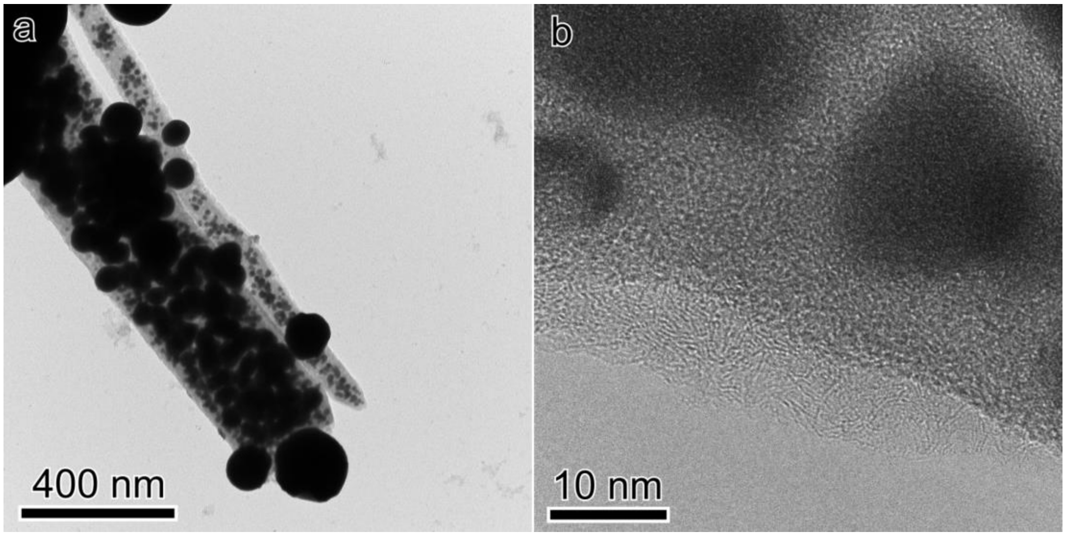

Figure S3. (a) TEM and (b) HRTEM images of Bi NPs@NC.

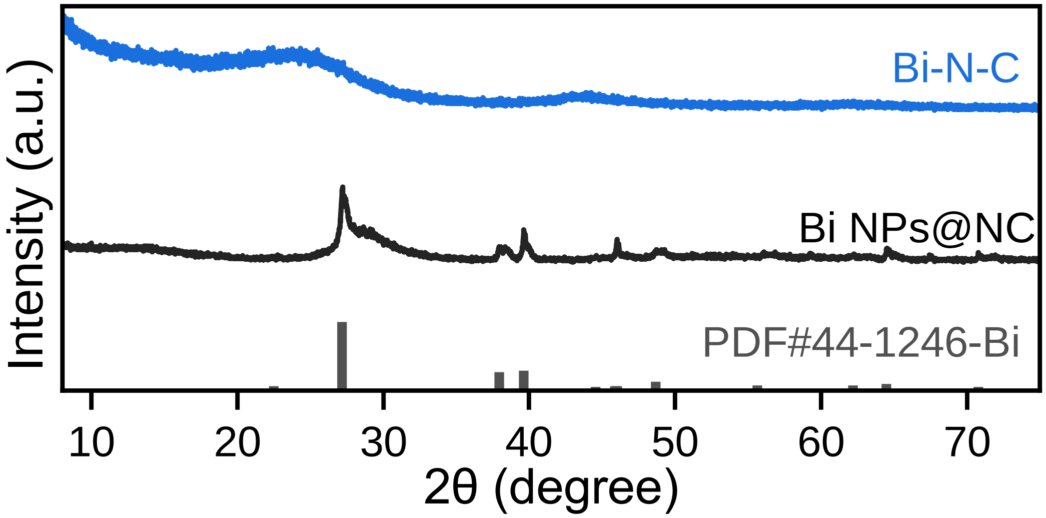

Figure S4. Measured XRD patterns of Bi NPs@NC and Bi-N-C. The theoretical pattern of bismuth metal is shown for comparison.

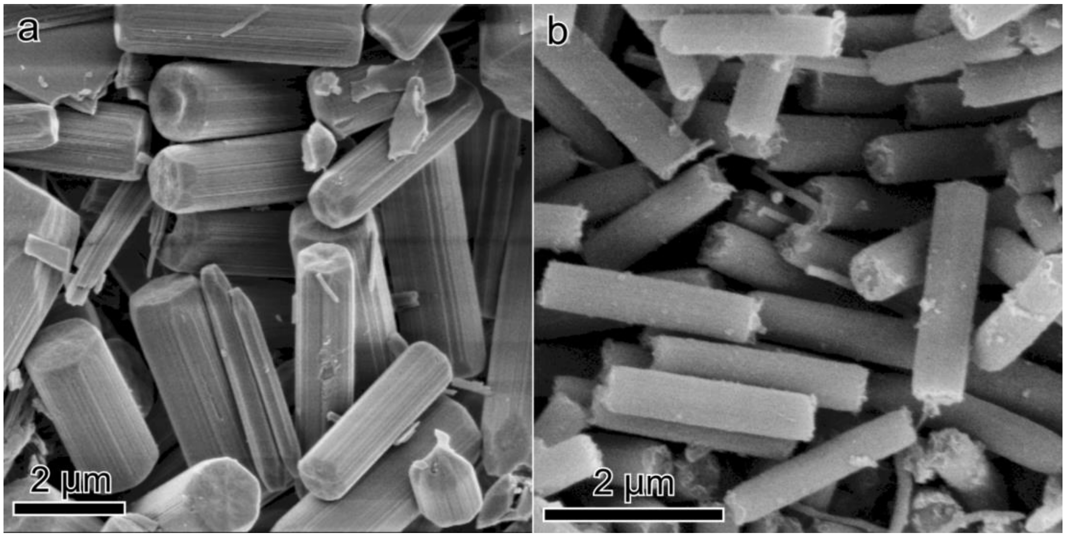

Figure S5. SEM images of (a) Bi-MOM and (b) Bi-N-C.

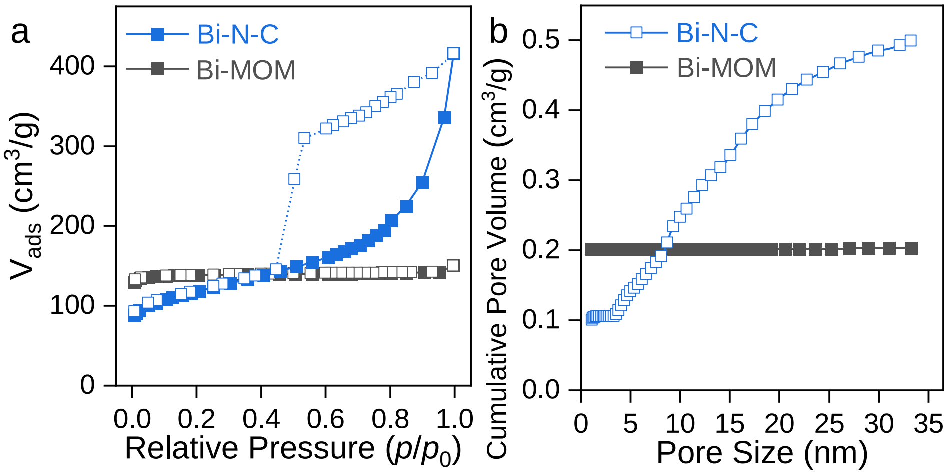

Figure S6. (a) Nitrogen physisorption isotherms and (b) corresponding pore size distributions of Bi-MOM and Bi-N-C.

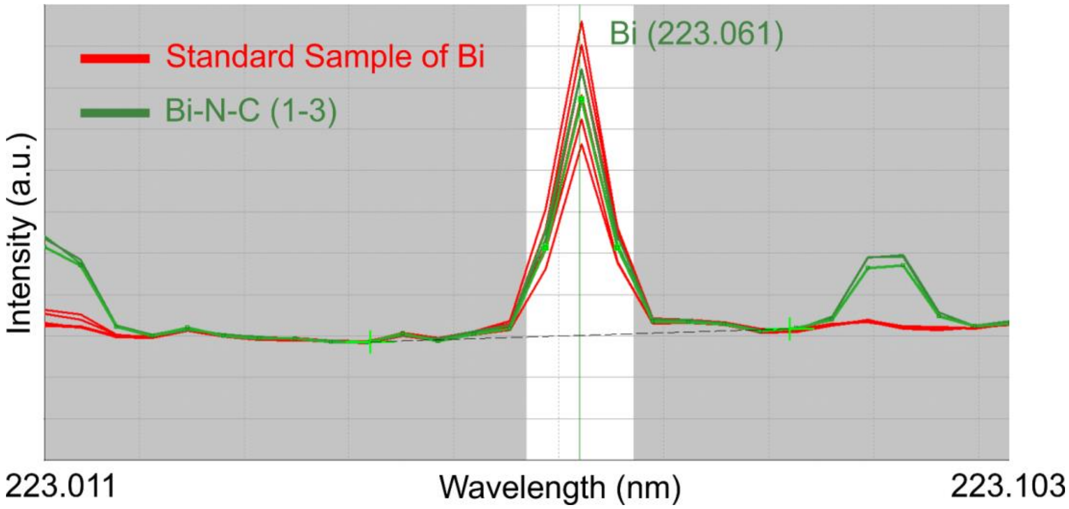

Figure S7. ICP-OES emission of standard sample of bismuth and Bi-N-C.

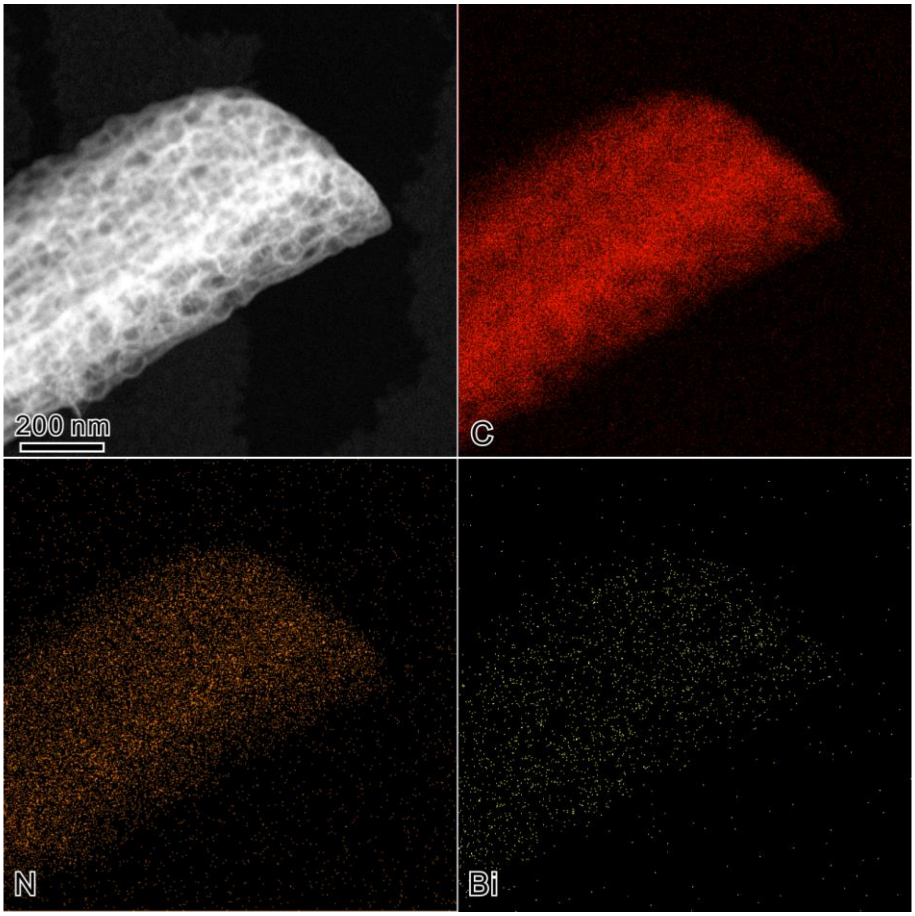

Figure S8. EDX-mapping results of Bi-N-C.

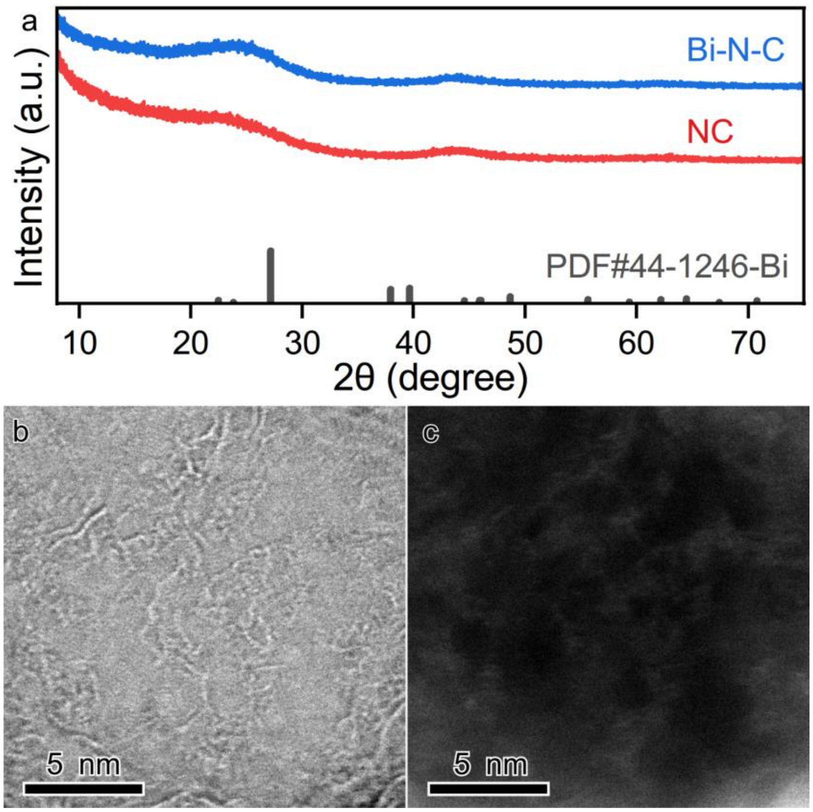

Figure S9. (a) XRD patterns of NC and Bi-N-C. (b) HRTEM and (c) AC HAADF-STEM images of NC.

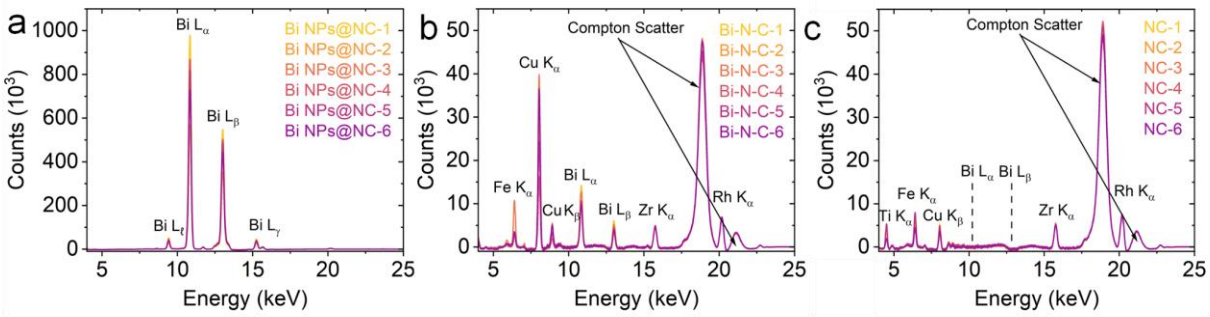

Figure S10. µXRF spectra of (a) Bi NPs@NC, (b) Bi-N-C and (c) NC of 6 individual point measurements. A different y-scale was used in a) because signal intensity of Bi is approximately 100-fold higher than in b) and c). As a consequence, signals of elements at much lower concentration are not clearly visible in a). Further elements detected: Zr signal results from the experimental set-up, namely from the detector aperture; Rh signal results from the X-ray excitation source; Fe, Cu are ubiquitous and are present as impurities within the materials and/or sample carrier;

Ti was detected only in NC and may be present as an impurity in the blank material.

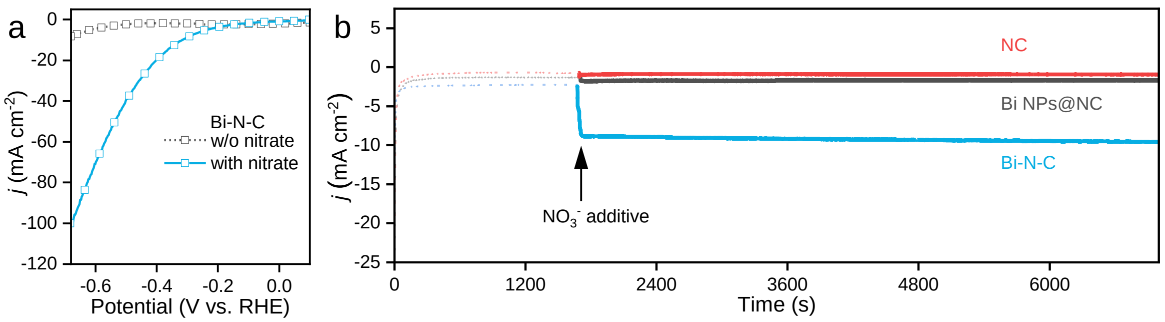

Figure S11. (a) LSV curves and (b) Nitrate-response chrono-amperometry results of Bi NPs@NC, Bi-N-C and NC at -0.35 V vs. RHE.

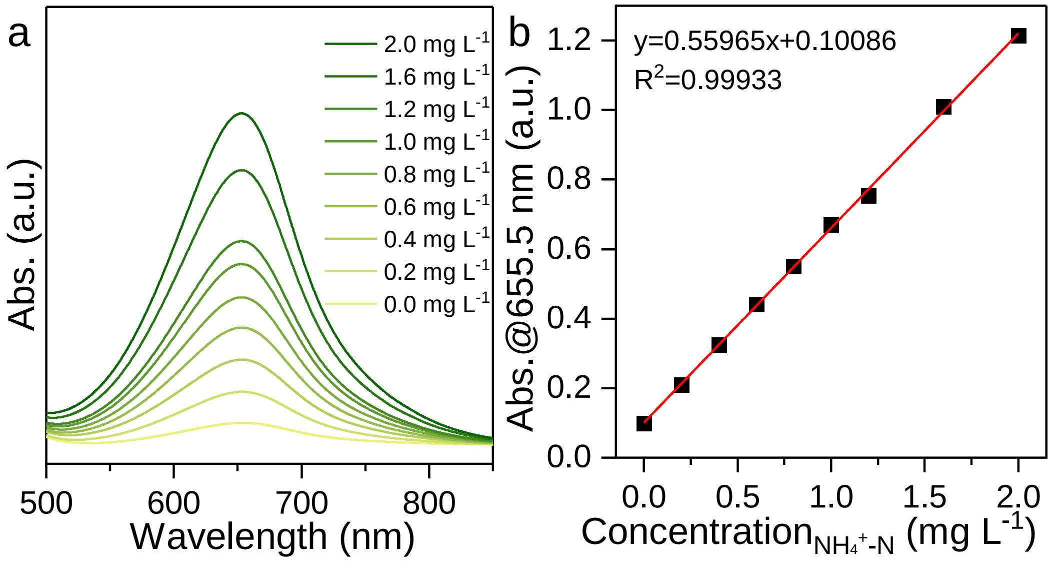

Figure S12. (a) UV-vis spectra with the absorbance of indophenol blue at 655.5 nm at different concentration of ammonium and (b) corresponding calibration curve used for NH_4^+ calculation.

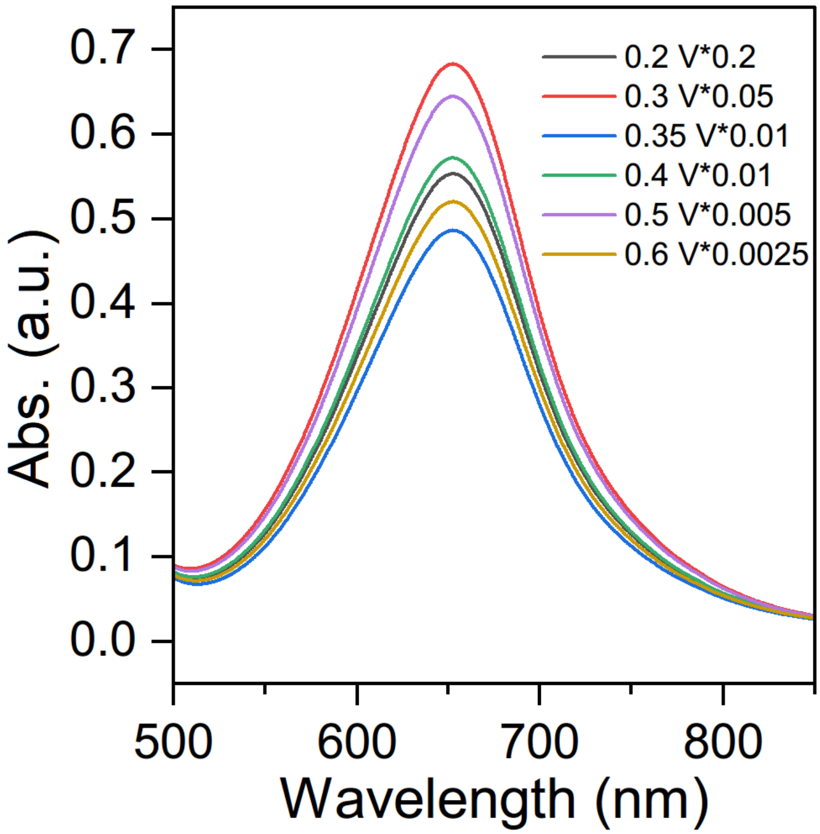

Figure S13. UV-vis spectra of electrolytes measured by the indophenol-blue method after NARR (the electrolyte has been diluted with different factors for testing.)

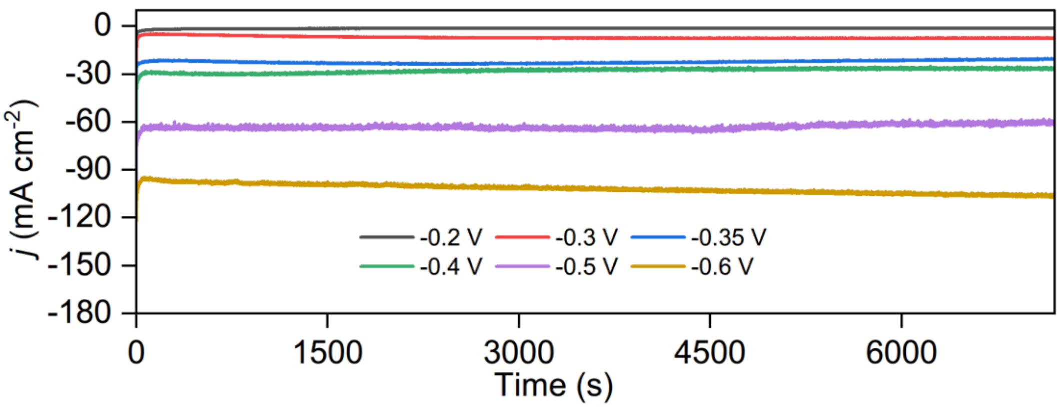

Figure S14. Chrono-amperometry results of Bi-N-C at different given potentials.

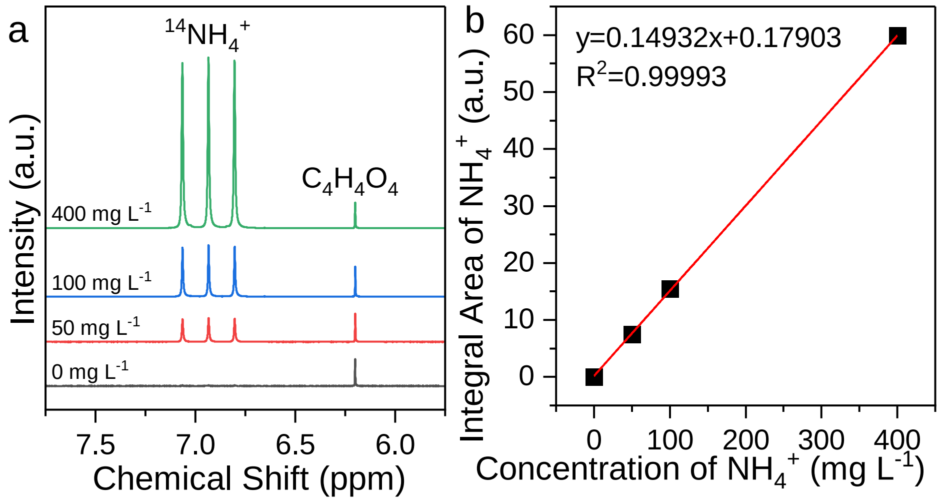

Figure S15. (a) ^{1}H NMR spectra of NH_4^+ at different concentrations using maleic acid as an internal standard and (b) corresponding NMR calibration curve for quantification of NH_4^+ in solution.

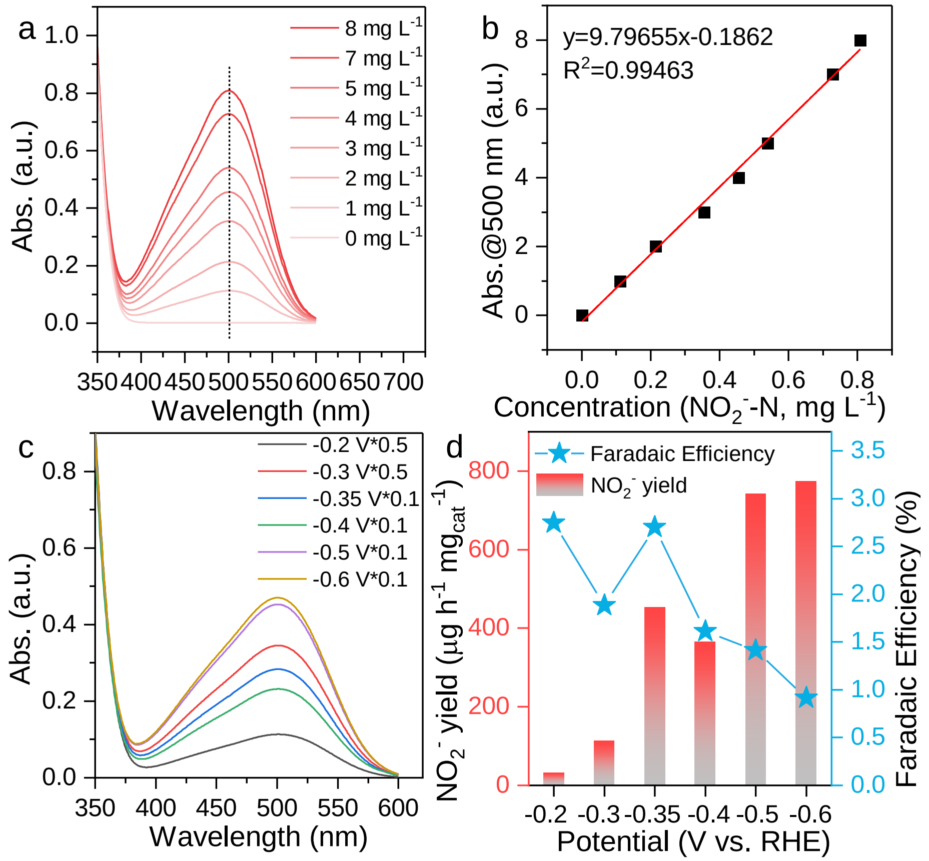

Figure S16. (a) UV-vis spectroscopy curves and (b) corresponding calibration curve used for NO_2^--N calculation. (c) UV-vis spectroscopy of electrolytes under all given potentials and (d) the corresponding FE as well as nitrite yield.

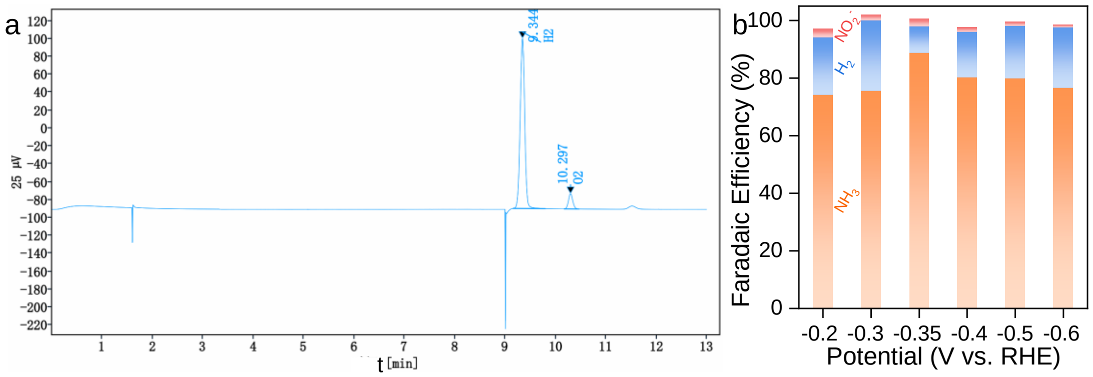

Figure S17. (a) Gas chromatrography measurements of Bi-N-C at -0.35 V vs. RHE. The peak at a retention time of 9.34 min indicates the formation of H_2. (b) The FEs for ammonia, hydrogen and nitrite at all given potentials.

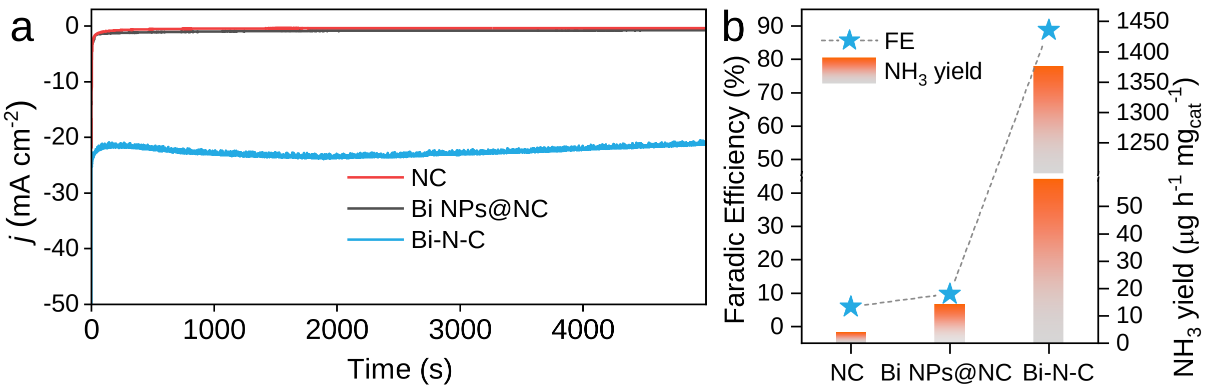

Figure S18. Comparison of (a) Chrono-amperometry results, (b) FE and ammonia yield results at -0.35 V vs. RHE between Bi-N-C, Bi NPs@NC and NC.

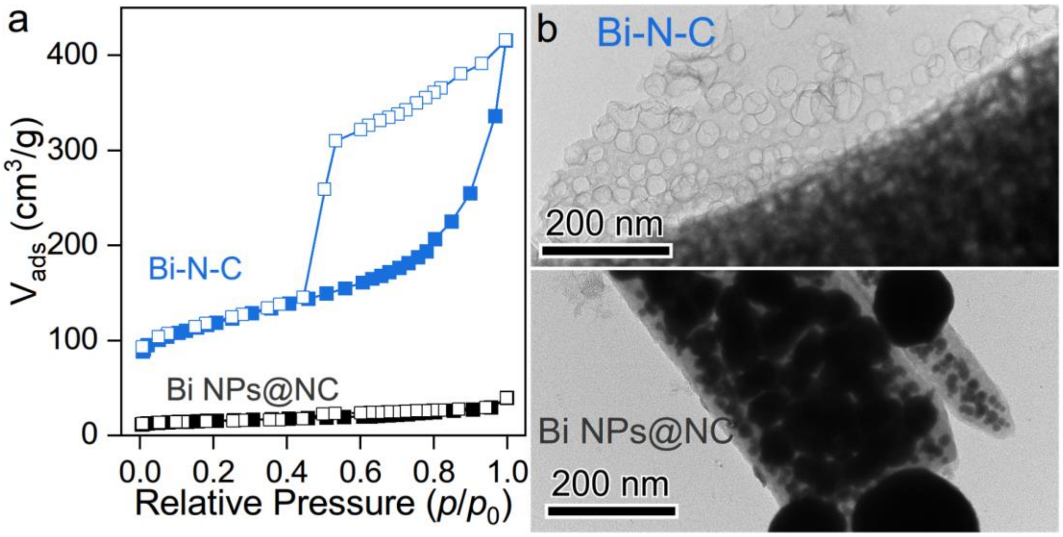

Figure S19. (a) Nitrogen physisorption isotherms and (b) TEM comparison of Bi-N-C and Bi NPs@NC.

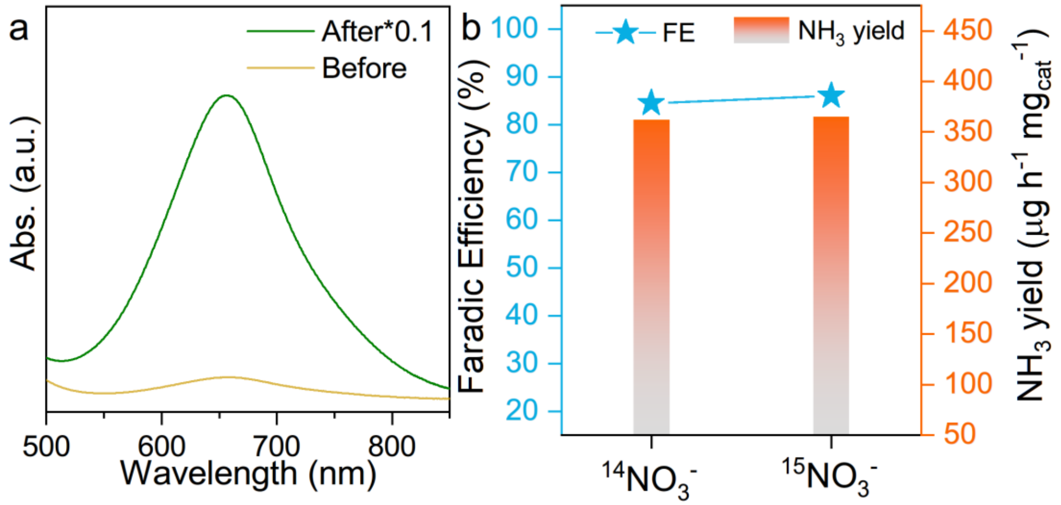

Figure S20. (a) UV-vis spectra of the electrolyte before and after NARR with $^{15}NO_3^-$ as reactant. (b) FE and ammonia yield comparison between different nitrogen sources (the electrolyte here is 0.5 M KOH with 50 mM $K^{14/15}NO_3$).

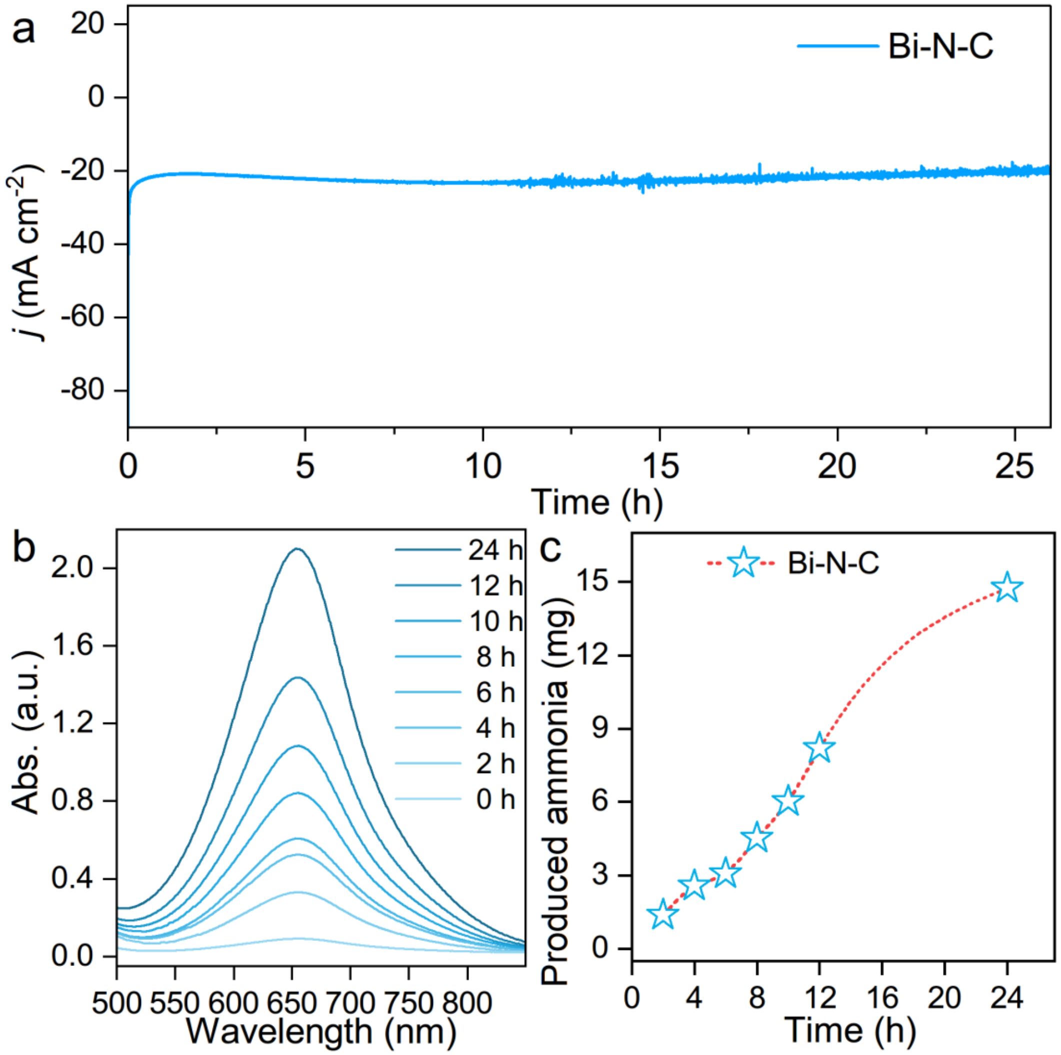

Figure S21. (a) Chrono-amperometry curve during the 24 h test, (b) UV-vis spectra (dilution factor is 800 here for every point), and (c) corresponding cumulative ammonia production after different times.

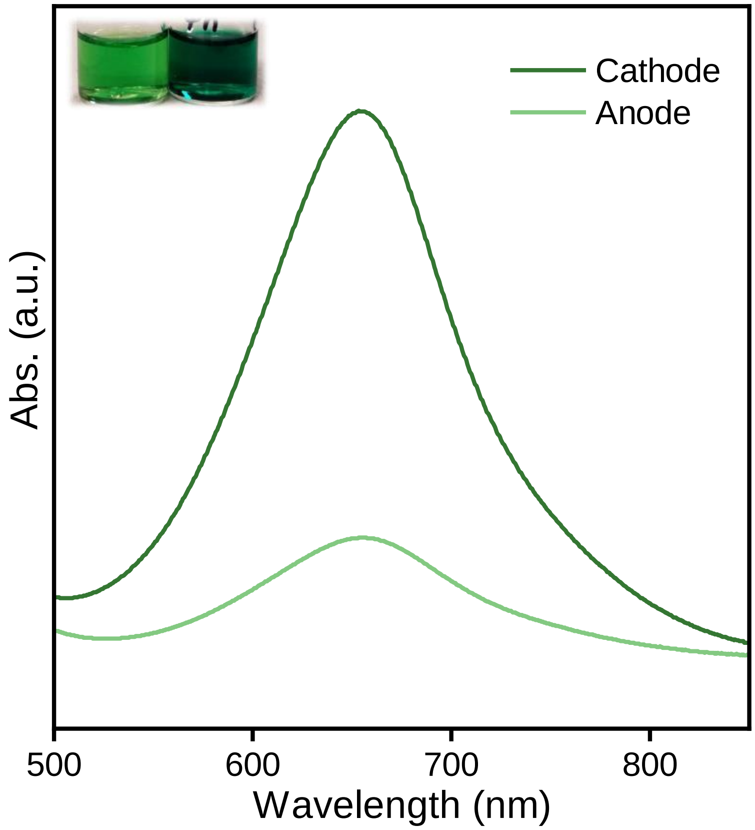

Figure S22. UV-vis spectra of electrolyte extracted from cathode and anode after 24-h electrolysis (dilution factor is 800 here for both chamber).

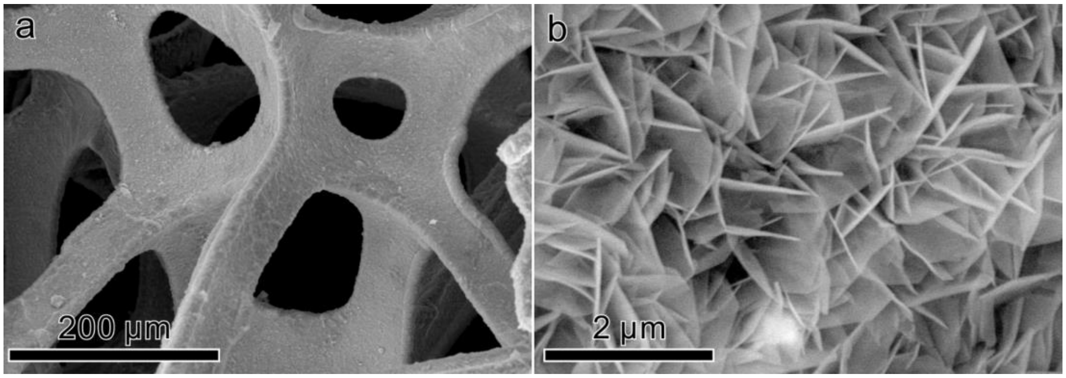

Figure S23. The SEM images of Ni-Fe LDH on nickel foam.

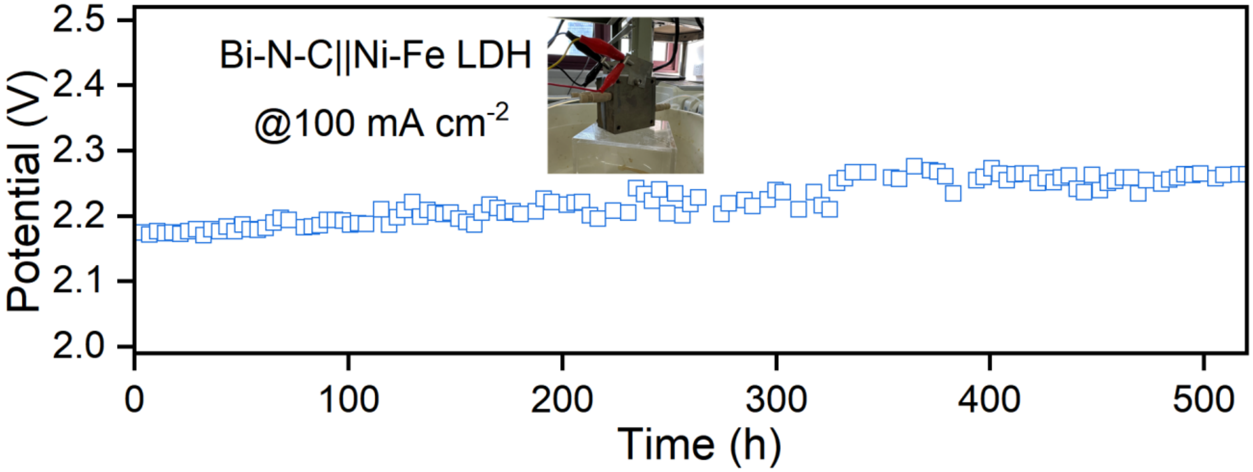

Figure S24. The chronopotentiometry curve of Bi-N-C||Ni-Fe LDH. (The insert picture is the constructed device)

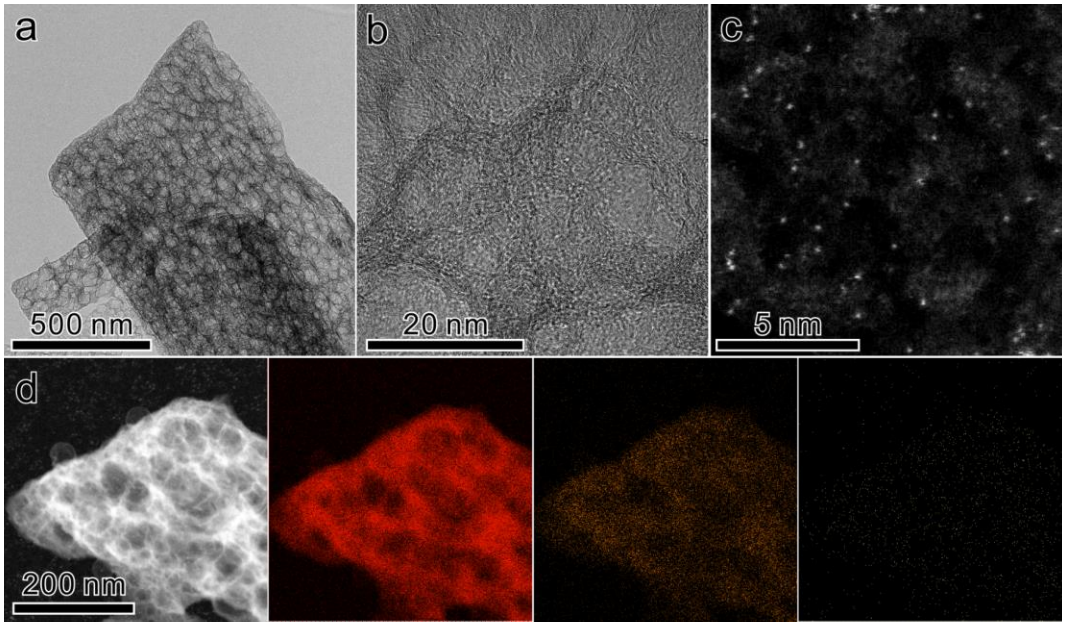

Figure S25. (a, b) TEM, (c) AC HAADF-STEM and (d) EDX mapping images of Bi-N-C after long-term stability test.

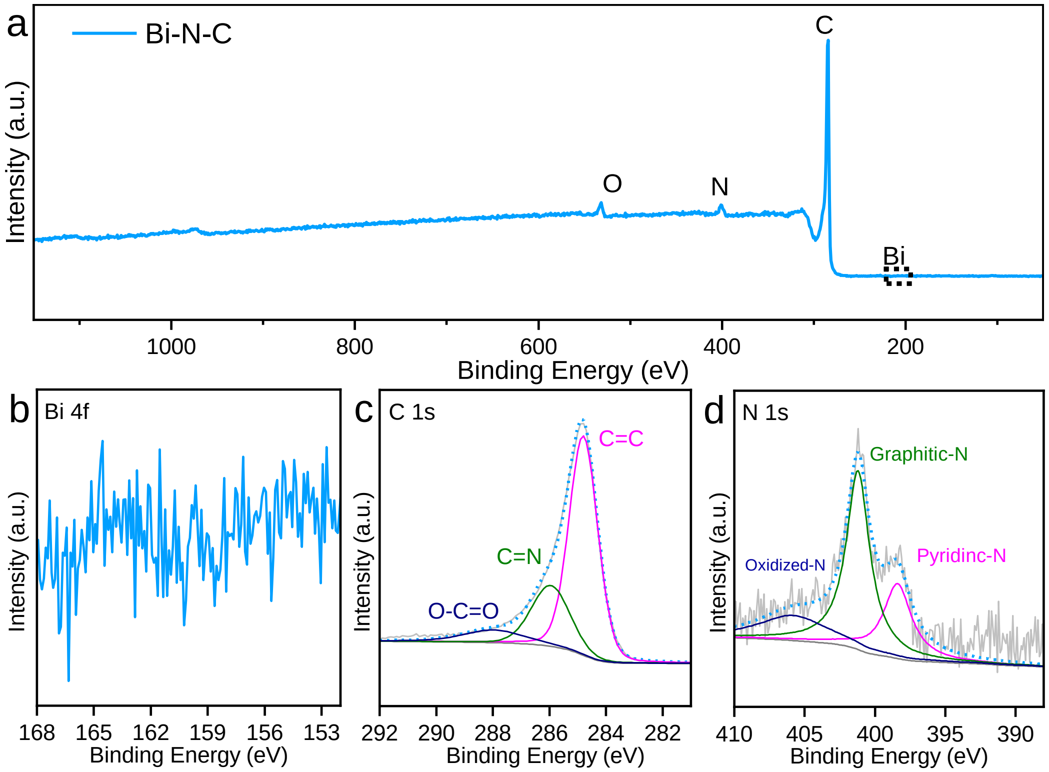

Figure S26. (a) XPS survey of Bi-N-C. High resolution (b) Bi 4f, (c) C 1s and (d) N 1s spectrum of Bi-N-C.

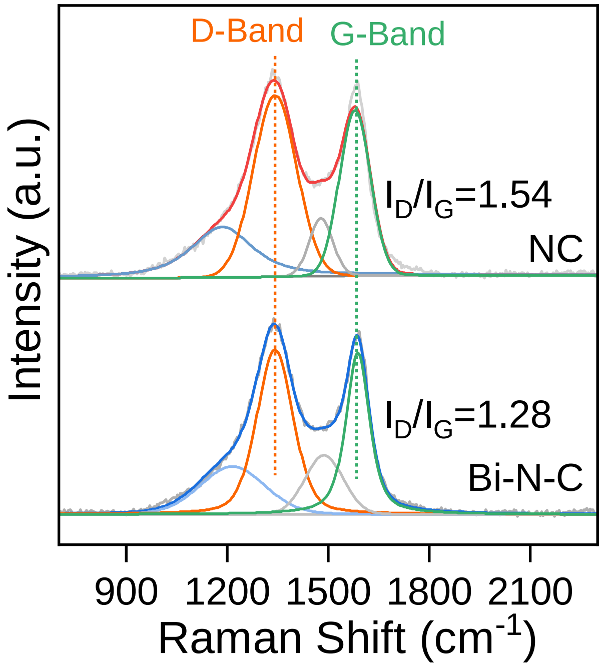

Figure S27. Raman spectra of NC and Bi-N-C.

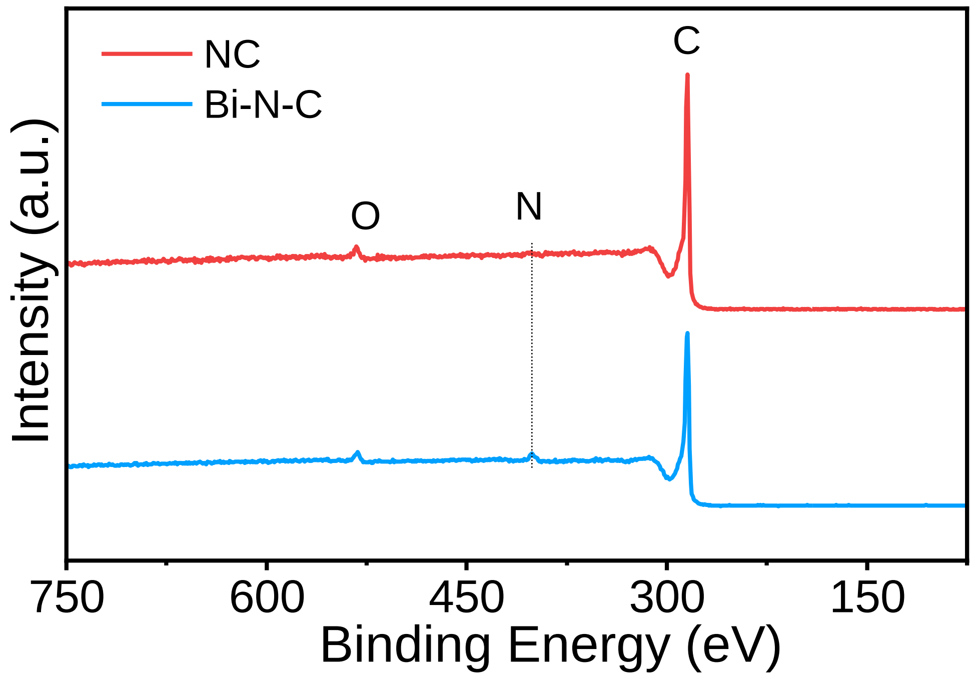

Figure S28. XPS survey spectra of Bi-N-C and NC.

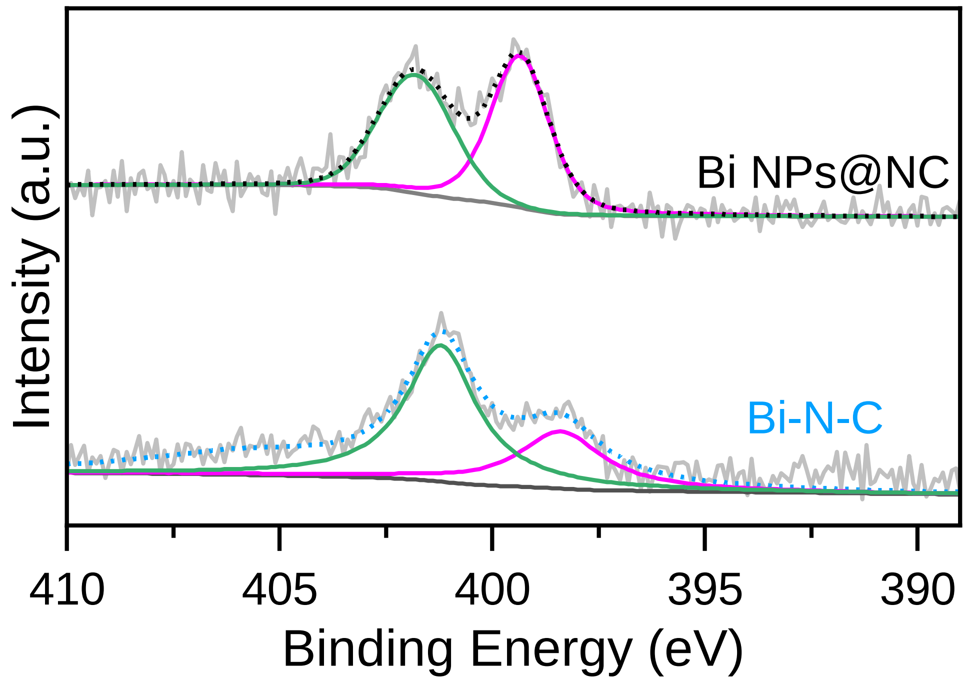

Figure S29. The high-resolution N 1s XPS spectra of Bi-N-C and Bi NPs@NC.

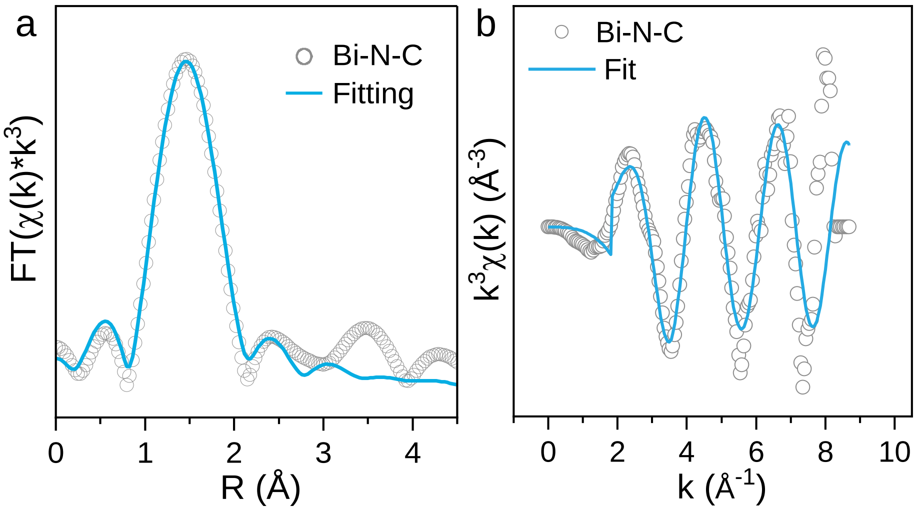

Figure S30. (a) EXAFS R-space and (b) k-space fitting results of Bi-N-C.

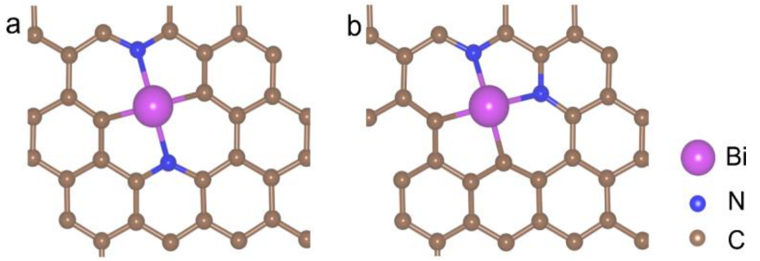

Figure S31. The DFT optimized structures for the two models of Bi-N-C. The calculated electronic energy for model a is 0.11eV lower than that of model b.

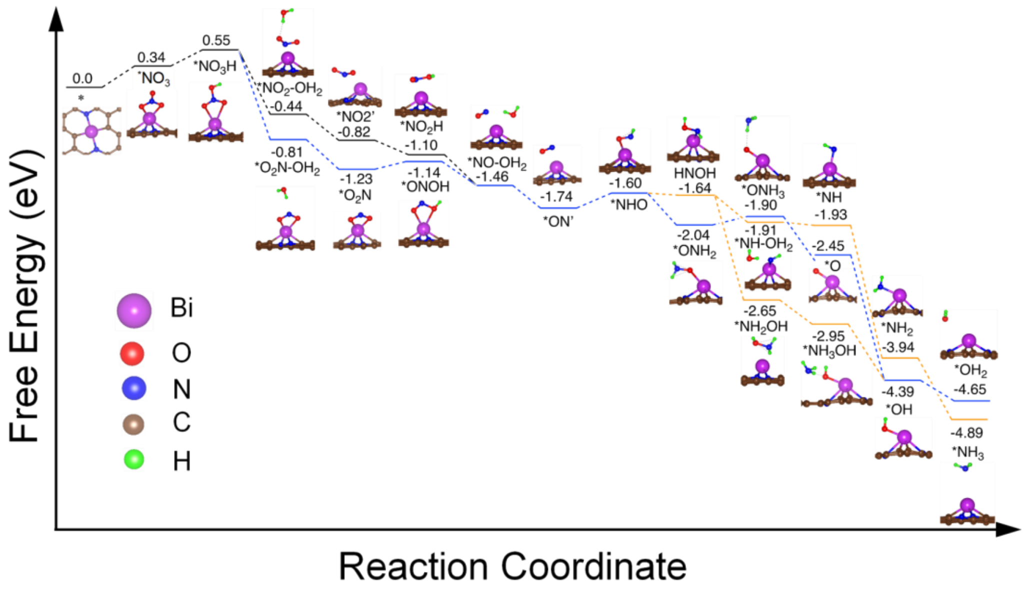

Figure S32. The energy panel of the reaction pathway on the BiN_2C_2 model.

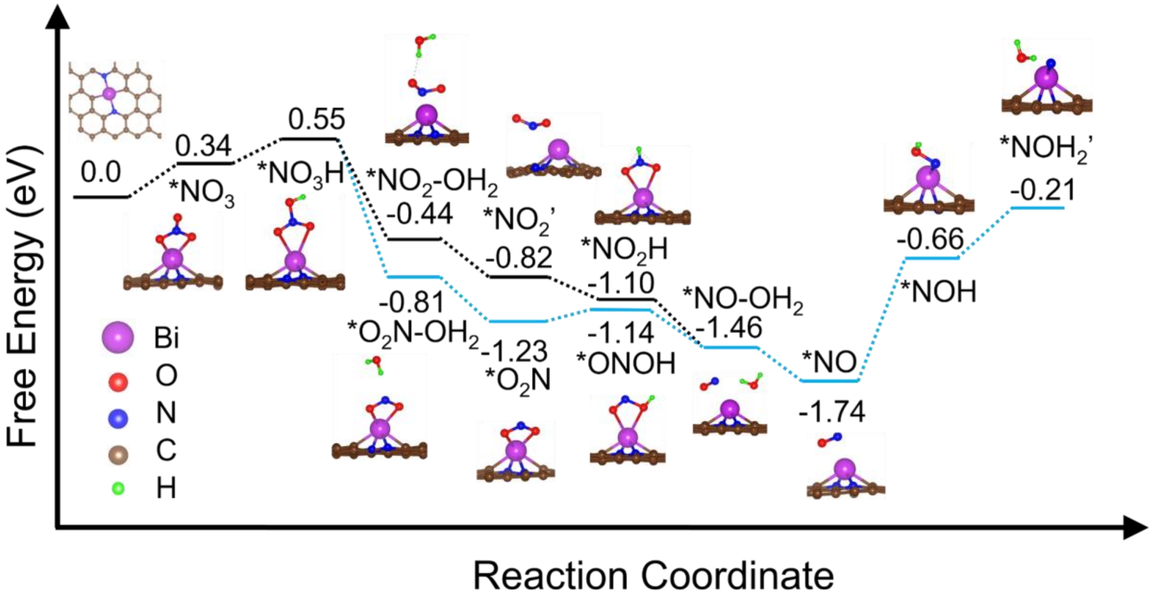

Figure S33. The energy panel of the reaction pathway on the BiN_2C_2 model with hydrogenation on the oxygen atom of the NO intermediate.

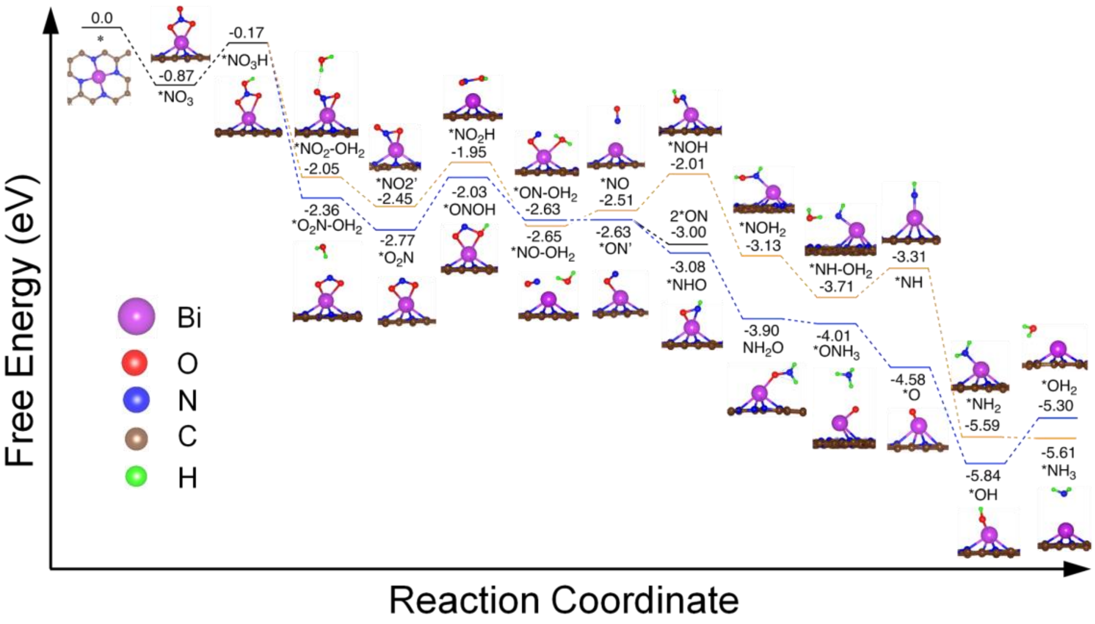

Figure S34. The energy panel of the reaction pathway on the BiN_4 model.

Table S1. The performance comparison of Bi-N-C and other reported catalysts.

Catalyst	NH_3 yield Rate	FE	Current density (mA cm^{-2})	Ref
Strained Ru NCs	1.17 mmol h^{-1} cm^{-2}	96%	120	[12]
$Ru_{15}Co_{85}$	3.21 mol h^{-1} g_{cat}^{-1}	97%	100	[13]
Ru dispersed Cu NWs	7000 μg h^{-1} cm^{-2}	93%	1000	[14]
Amorphous Ru NCs	145.1 μg h^{-1} mg_{cat}^{-1}	80.62%	4.5	[15]
Ru_xO_y clusters	274 μg h^{-1} mg_{cat}^{-1}	73%	27.5	[16]
$Cu_{50}Ni_{50}$	Not mentioned	93%	50	[17]
O-Cu-PTCDA	436 μg h^{-1} cm^{-2}	77%	15	[18]
CuCo nanosheets	1.17 mmol h^{-1} cm^{-2}	100%	1035	[19]
Co NAs	4.16 mmol h^{-1} cm^{-2}	96%	250	[20]
Co-Fe@Fe_2O_3	1.5 mg h^{-1} cm^{-2}	85%	10	[21]
FeB_2	25.5 mg h^{-1} cm^{-2}	96.8%	320	[22]
$Fe_2Ti_2O_5$ NFs	0.73 mmol h^{-1} mg_{cat}^{-1}	87.6%	10	[23]
Fe SAC	0.46 mmol h^{-1} cm^{-2}	75%	35	[24]
Cu SAC	4.5 mg h^{-1} cm^{-2}	84.7%	15	[25]
FeN_2O_2 SAC	46 mg h^{-1} mg_{cat}^{-1}	92%	Not mentioned	[26]
**Our work**	**1.38 mg h^{-1} mg_{cat}^{-1}**	**88.7**	**20**	/

Table S2. EXAFS fitting results for Bi-N-C at the Bi L_3-edge (S_0^2=0.36) C.N: co-ordination numbers; R: bond distance; σ^2: Debye-Waller factors; d ΔE: the inner potential correction. R factor: goodness of fit.

Sample	Path	C.N.	R (Å)	$\sigma^2 \times 10^3$ ($Å^2$)	ΔE (eV)	R factor
Bi_2O_3	Bi-O	6*	2.11±0.01	9.7±2.0	-3.9±1.2	0.003
Bi-N-C	Bi-N	2*	1.94±0.02	4.9±2.5	13.0±0.1	0.006
	Bi-C	2*	1.99±0.16			
Bi-N-C	Bi-N	4.5±0.9	2.09±0.2	11.9±3.4	-6.4±2.1	0.008

References

[1] P. Deng, F. Yang, Z. Wang, S. Chen, Y. Zhou, S. Zaman, B. Y. Xia, *Angew. Chem. Int. Ed.* **2020**, *59*, 10807-10813.

[2] a) C. Schlesiger, S. Praetz, R. Gnewkow, W. Malzer, B. Kanngiesser, *J. Anal. At. Spectrom.* **2020**, *35*, 2298-2304; b) C. Schlesiger, L. Anklamm, H. Stiel, W. Malzer, B. Kanngiesser, *J. Anal. At. Spectrom.* **2015**, *30*, 1080-1085.

[3] B. Ravel, M. Newville, **2005**.

[4] A. Sadezky, H. Muckenhuber, H. Grothe, R. Niessner, U. Pöschl, *Carbon* **2005**, *43*, 1731-1742.

[5] L. Zhou, C. E. Boyd, *Aquaculture* **2016**, *450*, 187-193.

[6] R. Y. Hodgetts, A. S. Kiryutin, P. Nichols, H.-L. Du, J. M. Bakker, D. R. Macfarlane, A. N. Simonov, *ACS Energy Lett.* **2020**, *5*, 736-741.

[7] Y. Wang, Y. Yu, R. Jia, C. Zhang, B. Zhang, *Natl. Sci. Rev.* **2019**, *6*, 730-738.

[8] a) G. Kresse, J. Furthmuller, *Comput. Mater. Sci.* **1996**, *6*, 15-50; b) G. Kresse, J. Furthmuller, *Phys. Rev. B Condens. Matter* **1996**, *54*, 11169-11186.

[9] P. E. Blochl, *Phys. Rev. B Condens. Matter.* **1994**, *50*, 17953-17979.

[10] J. P. Perdew, K. Burke, M. Ernzerhof, *Phys. Rev. Lett.* **1996**, *77*, 3865-3868.

[11] S. Grimme, J. Antony, S. Ehrlich, H. Krieg, *J. Chem. Phys.* **2010**, *132*, 154104.

[12] J. Li, G. Zhan, J. Yang, F. Quan, C. Mao, Y. Liu, B. Wang, F. Lei, L. Li, A. W. M. Chan, L. Xu, Y. Shi, Y. Du, W. Hao, P. K. Wong, J. Wang, S. X. Dou, L. Zhang, J. C. Yu, *J. Am. Chem. Soc.* **2020**, *142*, 7036-7046.

[13] S. Han, H. Li, T. Li, F. Chen, R. Yang, Y. Yu, B. Zhang, *Nat. Catal.* **2023**.

[14] F. Y. Chen, Z. Y. Wu, S. Gupta, D. J. Rivera, S. V. Lambeets, S. Pecaut, J. Y. T. Kim, P. Zhu, Y. Z. Finfrock, D. M. Meira, G. King, G. Gao, W. Xu, D. A. Cullen, H. Zhou, Y. Han, D. E. Perea, C. L. Muhich, H. Wang, *Nat Nanotechnol* **2022**, *17*, 759-767.

[15] M. Jiang, A. Tao, Y. Hu, L. Wang, K. Zhang, X. Song, W. Yan, Z. Tie, Z. Jin, *ACS Appl. Mater. Interfaces* **2022**, *14*, 17470-17478.

[16] J. Qin, K. Wu, L. Chen, X. Wang, Q. Zhao, B. Liu, Z. Ye, *J. Mater. Chem. A* **2022**, *10*, 3963-3969.

[17] Y. Wang, A. Xu, Z. Wang, L. Huang, J. Li, F. Li, J. Wicks, M. Luo, D.-H. Nam, C.-S. Tan, Y. Ding, J. Wu, Y. Lum, C.-T. Dinh, D. Sinton, G. Zheng, E. H. Sargent, *J. Am. Chem. Soc.* **2020**, *142*, 5702-5708.

[18] G. F. Chen, Y. F. Yuan, H. F. Jiang, S. Y. Ren, L. X. Ding, L. Ma, T. P. Wu, J. Lu, H. H. Wang, *Nat. Energy* **2020**, *5*, 605-613.

[19] J.-Y. Fang, Q.-Z. Zheng, Y.-Y. Lou, K.-M. Zhao, S.-N. Hu, G. Li, O. Akdim, X.-Y. Huang, S.-G. Sun, *Nat. Commun.* **2022**, *13*, 7899.

[20] X. Deng, Y. Yang, L. Wang, X. Z. Fu, J. L. Luo, *Adv Sci* **2021**, *8*, 2004523.

[21] S. Zhang, M. Li, J. Li, Q. Song, X. Liu, *Proc. Natl. Acad. Sci.* **2022**, *119*, e2115504119.

[22] G. Zhang, X. Li, K. Chen, Y. Guo, D. Ma, K. Chu, *Angew. Chem. Int. Ed.* **2023**, *62*, e202300054.

[23] H. Du, H. Guo, K. Wang, X. Du, B. A. Beshiwork, S. Sun, Y. Luo, Q. Liu, T. Li, X. Sun, *Angew. Chem. Int. Ed.* **2023**, e202215782.

[24] Z. Y. Wu, M. Karamad, X. Yong, Q. Huang, D. A. Cullen, P. Zhu, C. Xia, Q. Xiao, M. Shakouri, F. Y. Chen, J. Y. T. Kim, Y. Xia, K. Heck, Y. Hu, M. S. Wong, Q. Li, I. Gates, S. Siahrostami, H. Wang, *Nat. Commun.* **2021**, *12*, 2870.

[25] J. Yang, H. Qi, A. Li, X. Liu, X. Yang, S. Zhang, Q. Zhao, Q. Jiang, Y. Su, L. Zhang, J.-F. Li, Z.-Q. Tian, W. Liu, A. Wang, T. Zhang, *J. Am. Chem. Soc.* **2022**, *144*, 12062-12071.

[26] W.-D. Zhang, H. Dong, L. Zhou, H. Xu, H.-R. Wang, X. Yan, Y. Jiang, J. Zhang, Z.-G. Gu, *Appl. Catal. B. Environ* **2022**, *317*, 121750.
